# Pooled prevalence of lymphopenia in all-cause hospitalisations and association with infection: a systematic review and meta-analysis

**DOI:** 10.1186/s12879-023-08845-1

**Published:** 2023-12-02

**Authors:** ZC Elçioğlu, L Errington, B Metes, W Sendama, J Powell, AJ Simpson, AJ Rostron, TP Hellyer

**Affiliations:** 1 Translational and Clinical Research Institute, Faculty of Medical Sciences, Newcastle University, Newcastle-upon-Tyne, NE2 4HH UK; 2 Faculty of Medical Sciences Library, Newcastle University, Newcastle-upon-Tyne, NE2 4HH UK; 3 Department of Respiratory Medicine, Royal Victoria Infirmary, Newcastle-upon-Tyne, NE1 4LP UK; 4 Integrated Critical Care Unit, Sunderland Royal Hospital, South Tyneside and Sunderland NHS Foundation Trust, Sunderland, SR4 7TP UK; 5 Department of Critical Care Medicine, Royal Victoria Infirmary, Newcastle-upon-Tyne, NE1 4LP UK

**Keywords:** Prevalence, Lymphopenia, Infection, Healthcare-associated infection, Systematic review, Meta-analysis

## Abstract

**Background:**

Lymphopenia is defined as a decrease below normal value (often 1.0 x 10^9^ cells/L) of blood circulating lymphocyte count. In the general population, lymphopenia is associated with an increased risk of hospitalisation secondary to infection, independent of traditional clinical risk factors. In hospital, lymphopenia is associated with increased risk of healthcare-associated infection and mortality. By summarising lymphopenia’s prevalence and impact on clinical outcomes, we can identify an at-risk population and inform future studies of immune dysfunction following severe illness.

**Methods:**

Peer-reviewed search strategy was performed on three databases. Primary objective was to summarise the pooled prevalence of lymphopenia. Primary outcome was infection including pre-existing lymphopenia as a risk factor for admission with infection and as an in-hospital risk factor for healthcare-associated infection. Secondary outcomes were length of stay and mortality. Mortality data extracted included in-hospital, 28/30-day (‘early’), and 90-day/1-year (‘late’) mortality. Meta-analysis was carried out using random-effects models for each outcome measure. Heterogeneity was assessed using I^2^ statistic. Joanna Briggs Institute checklist for cohort studies was used to assess risk of bias. The protocol was published on PROSPERO.

**Results:**

Fifteen observational studies were included. The pooled prevalence of lymphopenia in all-cause hospitalisations was 38% (CI 0.34-0.42, I^2^= 97%, *p< 0.01)*. Lymphopenia was not associated with an infection diagnosis at hospital admission and healthcare associated infection (RR 1.03; 95% CI 0.26-3.99, *p=0.97*, I^2^ = 55% and RR 1.31; 95% CI 0.78-2.20, *p=0.31*, I^2^=97%, respectively), but was associated with septic shock (RR 2.72; 95% CI 1.02-7.21, *p=0.04*, I^2^ =98%). Lymphopenia was associated with higher in-hospital mortality and higher ‘early’ mortality rates (RR 2.44; 95% CI 1.71-3.47, *p < 0.00001*, I^2^ = 89% and RR 2.05; 95% CI 1.64-2.56, *p < 0.00001,* I^2^ = 29%, respectively). Lymphopenia was associated with higher ‘late’ mortality (RR 1.59; 1.33-1.90, *p < 0.00001,* I^2^ = 0%).

**Conclusions:**

This meta-analysis demonstrates the high prevalence of lymphopenia across all-cause hospitalisations and associated increased risk of septic shock, early and late mortality. Lymphopenia is a readily available marker that may identify immune dysfunctional patients. Greater understanding of immune trajectories following survival may provide insights into longer-term poor clinical outcomes.

**Supplementary Information:**

The online version contains supplementary material available at 10.1186/s12879-023-08845-1.

## Background

Immune dysfunction plays a central role in a wide range of diseases including cancer, atherosclerosis, trauma, and infections [1–4]. In the context of sepsis, this dysfunction is demonstrated across innate and adaptive immunity, and is characterised by apoptosis of immune cells, dysfunction in cellular function of neutrophils and monocytes, and ‘T cell exhaustion’ [5–9]. The presence of these cellular dysfunctions is associated with poor clinical outcomes, including healthcare-associated infections, increased mortality, and prolonged hospital length of stay [10, 11]. Immune modulation in cancer through immune checkpoint blockade has revolutionised cancer treatment [12]. There is intense focus on investigating novel therapies to modulate the immune dysfunction in sepsis, in the hope of improving clinical outcomes [13].

There is no one test to identify patients with immune dysfunction and assays are highly specialised and not readily available in a hospital setting [14]. Lymphopenia is a window into the state of the immune system, available from routinely collected clinical data. Lymphopenia has been associated with increased mortality and healthcare-associated infection amongst patients with sepsis [15]. Adverse outcomes associated with lymphopenia have been recognised in a wider hospital setting, including in patients with pneumonia and following stroke [16, 17]. In the general population, lymphopenia is associated with an increased risk of hospitalisation secondary to infection, independent of clinical risk factors such as age and co-morbidities [18, 19].

The ability to identify and quantify this at-risk population is important for designing future studies to modulate the immune response and to investigate the longer-term impact of immune dysfunction in hospital. Since lymphopenia has been shown to lead to poor clinical outcomes in such a wide range of hospital populations, we sought to summarise the pooled prevalence of lymphopenia in hospitalised patients regardless of the cause of hospital admission. In addition, we aimed to determine the impact of lymphopenia on clinical outcomes including infection, mortality and length of hospital stay.

## Methods

### Protocol and registration

The systematic review was registered prospectively with PROSPERO (CRD42022327031) and was reported according to the Preferred Reporting Items for Systematic Reviews and Meta-Analysis (PRISMA) guidelines [20, 21].

### Study search strategy

PROSPERO and Cochrane Library searches confirmed that there were no previous systematic reviews of prevalence of lymphopenia in all-cause hospital admissions. Searches were performed on MEDLINE, Embase and CENTRAL databases. Studies that allowed extraction of prevalence data of lymphopenia were included. Records were not restricted by publication date. Records were extracted to Endnote (Thompson, Reuters, Philadelphia, PA, USA) to remove duplicate studies.

Rayyan was used for title and abstract screening [22]. A sample of 10% of results were reviewed by two reviewers to establish agreement (ZCE and TPH). Any disagreements were re-evaluated and resolved between the two reviewers. Data extraction was carried out by a single reviewer (ZCE).

### Exclusion criteria

Narrative reviews, editorials, case reports, duplicate publications, qualitative studies, conference abstracts, and non-human studies were excluded. Studies were limited to adult populations and those published in English language. A protocol amendment, prior to formal screening of search results/data extraction, was published on PROSPERO to exclude studies where the primary focus were patients with pre-existing immunosuppression, HIV or COVID-19. This review aimed to summarise lymphopenia in the general hospital population, rather than in patients with immunosuppression (for example, secondary to chemotherapy), in whom lymphopenia and subsequent infection risk are well recognised. Studies of COVID-19 patients were excluded because lymphopenia in this context has been summarised in a recent systematic review [23]. This protocol update did not require a secondary amendment of the search strategy, as negative searching was not carried out.

### Data collection process

Data were extracted from the selected papers onto a pre-formatted Excel worksheet (Microsoft, Redmond, WA, USA) containing the following characteristics: author and year of publication; country of origin and setting; study design; duration of study; demographics including age and sex; sequential organ failure assessment (SOFA) score; acute physiology and chronic health evaluation II (APACHE II) score; co-morbidities where available; and the definition of lymphopenia. Outcomes reported were extracted including healthcare-associated infection, all-cause mortality, and length of stay. The diagnostic criteria used for infection and healthcare-associated infection were also extracted.

The number of events was extracted for dichotomous outcomes. Means with standard deviation (SD) were extracted for continuous outcomes. Median values, interquartile ranges and sample size were used to estimate the sample mean and SD [24, 25].

### Outcomes

The primary objective was to summarise the pooled prevalence of lymphopenia in all-cause hospital admissions. The primary clinical outcome was infection, including infection at admission and healthcare-associated infection. Secondary clinical outcomes included length of hospital stay, length of intensive care unit (ICU) stay, all-cause in-hospital mortality, 28/30-day mortality (defined as ‘early’) and 90-day/1-year mortality (defined as ‘late’).

The definition of lymphopenia was determined by the paper being analysed in the review. Absolute lymphocyte count (ALC) is expressed in units of 10^9^/L. The normal range of ALC is often accepted to be between 1.5 to 4 x 10^9^/L.

### Risk of bias and quality of evidence assessment

The risk of bias was assessed by two reviewers (ZCE and TPH), using the Joanna Briggs Institute (JBI) critical appraisal checklist for observational studies [26].

An overall assessment of the evidence quality for outcome measures was reported according to the Grading of Recommendations, Assessment, Development and Evaluations (GRADE) assessment [27]. The software program GRADEpro was used [28].

### Statistical analysis

The meta-analysis was conducted using Review Manager and R Meta package [29–31]. A *p* value of less than 0.05 was accepted to be statistically significant. Dichotomous data were analysed using risk ratio (RR) with 95% confidence intervals (CIs). Continuous data were analysed using inverse variance (I-V) method to obtain mean difference (MD) and standard deviation (SD). Random-effects models for pooled analysis was used, independent of heterogeneity. Heterogeneity was assessed using the I^2^ statistic.

## Results

### Study selection

**Fig. 1:**
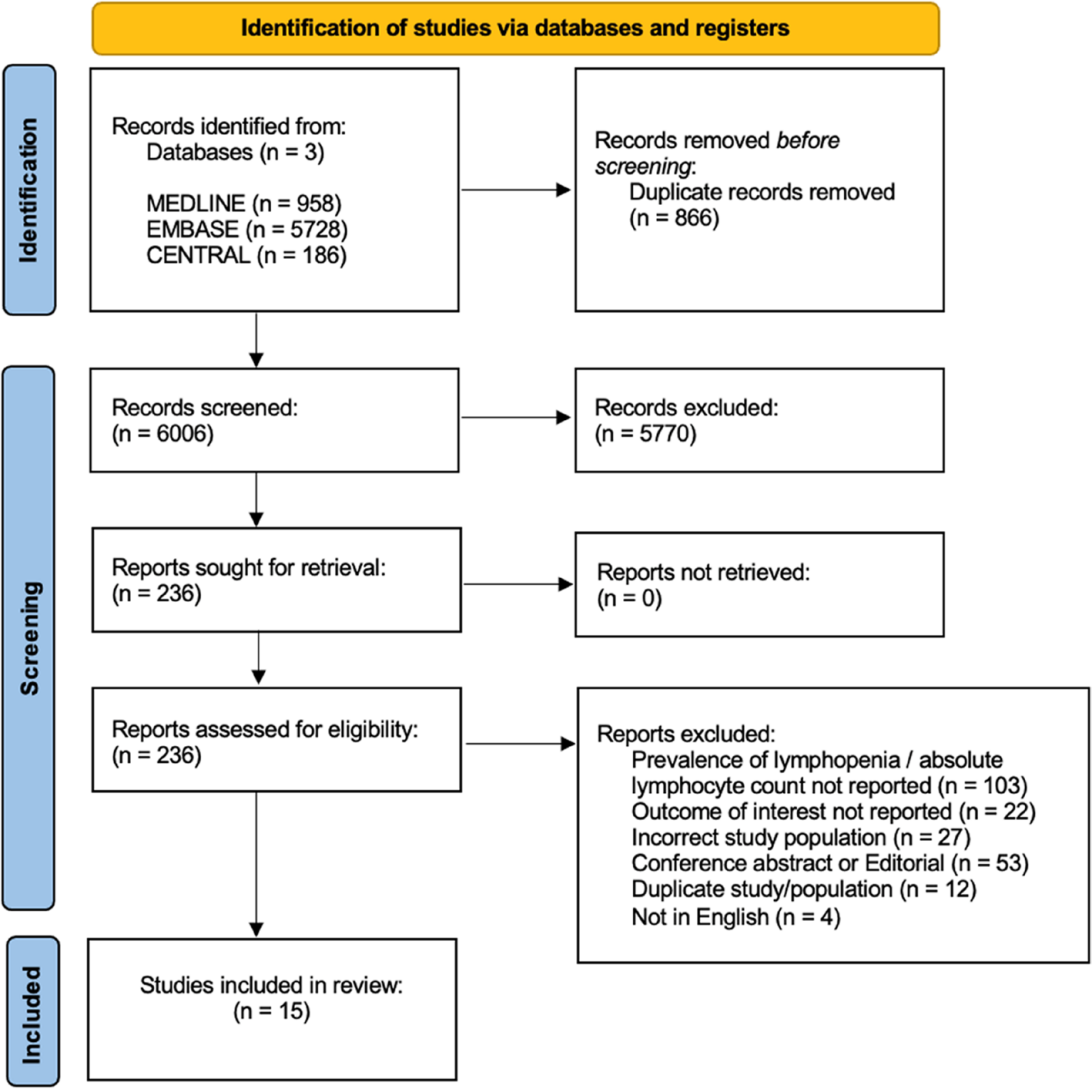
PRISMA flow diagram showing literature search results. Fifteen studies were used for meta-analysis. PRISMA, Preferred Reporting Items for Systematic Reviews and Meta-Analysis [21]

A total of 6006 studies were identified. After title and abstract screening, 236 potentially eligible studies underwent full-text review. The study flow diagram based on PRISMA guidelines demonstrates reasons for exclusion (Fig. 1). Following exclusion of 221 studies (Fig. 1), 15 studies were included in the analysis [15–17, 32–43].

### Study characteristics

A total of 72305 patients were included in the analysis (Table 1). Studies included a range of clinical conditions and settings, including elective admissions, critically ill patients, community-acquired pneumonia (CAP), ventilator-associated pneumonia (VAP), sepsis, spinal surgery, chest trauma, traumatic brain injury, and influenza A. Four studies were related to patients in ICUs only [34, 35, 40, 43].

**Table 1:** Characteristics of included papers. R* retrospective cohort study; P** prospective cohort study. *p* values*, where provided, are for comparisons between lymphopenic *vs* non-lymphopenic populations. HR, hazard ratio; OR, odds ratio; LOS, length of stay; IQR, interquartile range; RCT, randomised controlled trial; COPD, chronic obstructive pulmonary disease; UTI, urinary tract infection; CAP, community-acquired pneumonia; RT-PCR, reverse transcription polymerase chain reaction

**Study, Year** **Country** **Study Period** **Study Design**	**Population of Interest and Sample Size (n)**	**Lymphopenia Definition (10**^**9**^**/L)**	**Lymphopenic Prevalence (n, %)**	**Lymphopenic Population Characteristics [Age years, mean, SD] [Female n, %] [Co-morbidities n, %]**	**Timing of ALC**	**Outcome 1: Infection - cause of admission and/or nosocomial infection (definition) [n, %]**	**Outcome 2: Mortality [n, %] [OR, 95% Confidence Interval]**	**Outcome 3: Length of stay (LOS)- hospital or intensive care unit (ICU) [days, mean ± SD]**
**Andreu-Ballester *et al*. 2021 ** [32] Spain, 2 hospitals Jan 2016 to Dec 2019 R*	Patients older than 14 years and emergency and/or elective admission 58260	< 1.0	23892 (41%)	Age 73.5 ± 15.6 [p<0.001]* Female 10,905 (39.4)	Admission and any time during hospital stay	*Infection as cause of admission* 11016 (46.1)	*In-hospital mortality* Total deaths *n*= 3213 (5.5%) of which *n*=2345 (73%) had lymphopenia during hospital stay (OR 4.2, 3.9-4.6) Lymphopenia on admission 1743 (54.2%) (OR 2.8, 2.5-31)	N/A
**Bermejo-Martin *et al.* 2017 ** [16] Spain Derivation cohort (DC -multicentre 14 hospitals), January 2012 to June 2015 Validation cohort (VC - single centre) January 2005 to December 2015 R*	Presence of new pulmonary infiltrates on chest X-ray and respiratory signs/symptoms compatible with CAP DC 1550 VC 2846	<0.724 following subsequent AUC analysis (best cut-off value for identifying non-survivors)	DC 520 (33.5) VC 1014 (35.6)	Derivation Cohort Age>65 196 (37.7) Female 178 (34.2) Diabetes 113 (22.2) [p 0.915]* COPD 139 (27.3%) *[p 0.854]* Cardiac disease 176 (34.2%) *[*p*<0.001]* Liver disease 32 (6.3%) *[p 0.002]* Validation Cohort Age>65 644 (64.5) Female 370 (34.5) Diabetes 224 (22.6) [p 0.949]* COPD 350 (35.7) *[p 0.949]* Cardiac disease 129 (12.8) *[p 0.064]* Liver disease 45 (4.5) *[p 0.357]*	N/A	*N/A* *Septic shock subgroup:* DC 36 (6.9) VC 74 (7.5)	*30-day mortality* Derivation Cohort 41 (7.9) [p<0.001]* OR 1.93 (1.06-3.51)* [p 0.031]* Validation Cohort 116 (11.4) [p<0.001]* OR 1,86 (1.28-2.71)* [p 0.001]*	N/A
**Campbell *et al*. 2022 ** [33] USA, trauma centre hospital July 2009 to May 2018 R*	Traumatic brain injuries (concussion, subarachnoid, subdural, intraventricular, epidural, and intra-parenchymal haemorrhage) or diffuse axonal injury from blunt trauma Exclusion of death within 24 hours and/or bowel perforation on admission 2570	<1.0	946 (36.8)	Age 66.9 ± 23.0 Female 337 (39.9) Diabetes 68 (7.2) [ p 0.238]* COPD 58 (6.1) [ p 0.494]* Heart Failure 77(8.1 ) [ p 0.002]* Hypertension 495 (52.3) [ p <0.001]* Chronic liver disease 13 (1.4) [p 0.077]*	Within 24 hours of admission	Nosocomial Infection including pneumonia, UTI, septicaemia, intra-abdominal abscess and wound infection (*n*=380) of which 184 (48.4%) lymphopenic Pneumonia 84 (8.9) OR 1.510 (1.081-2.111) *[ p 0.016]* UTI 85 (9) OR 1.324 (0.960-1.826) *[p 0.087]*	*In-hospital mortality* 110 (11.6) [p<0.001]* Lymphopenia associated with increased risk of mortality OR = 1.903 (1.389-2.608) *[p<0.001]* Lymphopenic patients at higher risk of dying sooner in hospital OR 1.459 ( 1.097 - 1.941) *[p 0.009]*	Hospital LOS 5.7 ± 5.9 [p<0.001]* ICU LOS 5.1 ±5.2 [p 0.084]*
**Carneiro *et al.* 2021 ** [17] USA, neurocritical care unit November 2008 to April 2014 R*	Diagnosis of intracranial haemorrhage (including intra-ventricular haemorrhage and both supratentorial and infratentorial locations) on non-contrast CT scan 213	< 1.0 (or <1.1 depending on lab essay internal: validity at time of sample collection)	53 (24.9)	Age 69.0 ± 17.5 [p 0.082]* Female 22 (41.5) Hypertension 44 (83.02) [ p 0.5022]* Diabetes 15 (28.3) [p 0.9802]*	Admission	Nosocomial infection (i.e. 48 hours after admission). Subtypes included pneumonia, UTI, ventriculitis, endocarditis and bacteraemia Nosocomial Infection 23 (43.4) [p 0.037]* OR 2.15 (0.98- 4.73)* [p 0.0569]* Nosocomial UTI 13 (24.5) [ p 0.0033]* OR 3.66 (1.36, 9.88) *[p 0.0104]*	*In-hospital mortality* 18 (34.0) [p 0.0964]*	Hospital LOS 12.1 ± 15.2 [p 0.187]*
**Ceccato *et al.* 2019 ** [34] Spain, 6 ICUs in a single centre tertiary hospital (medical and surgical patients) 2005 to 2016 P**	≥ 18 years with clinical suspicion of pneumonia 48 h after ICU admission, with or without intubation and mechanical ventilation (IMV) 473	< 0.595	141 (29.8)	Age 67.3 ± 13.5 [p 0.13]* Female 42 (29.8) SOFA score 7 (5-10) Diabetes 40 (28) [p 0.12]* Chronic heart disorders 50 (35) [p 0.67]* COPD 38 (27) [p 0.063]* Chronic liver disease 39 (28) [p 0.002]*	Admission	N/A	*28-day mortality* 38 (27) [p 0.024]* *90-day mortality* 74 (53) [p<0.001]*	N/A
**Chung *et al.* 2015 ** [35] Taiwan, 2 hospitals October 2010 to January 2012 P**	≥20 years admitted to medical ICUs for severe sepsis or septic shock 92	< 0.5	24 (26.1)	Age 71.1 ± 13.8 [p 0.105]* Female 8 (39.1) SOFA score 11 (6.3-14) APACHE II score 21.5 (16.3-28.5) Congestive heart failure 2 (8.3) [p 1.00]* Diabetes 10 (41.7) [p 0.866]* Cirrhosis 3 (12.5) [p 0.180]*	Admission, day 1 and day 3 bloods	N/A	*28-day mortality* 13 (52.4)	N/A
**Drewry *et al.* 2014 ** [15] USA, tertiary hospital January 2010 to July 2012 R*	All adults admitted with sepsis and blood cultures positive for bacteria ± fungal organisms within 5 days of admission 335	<1.2 Severe lymphopenia ALC < 0.6 Moderate lymphopenia ALC 0.7 to 1.1	210 (62.7) Severe 76 Moderate 134	*Moderate persistent lymphopenia* Age 61.5 ± 16.1 [p 0.07]* Female 64 (47.8) APACHE II 17.6 (5.9) [p 0.002]* Congestive heart failure 37 (31.3) [p 0.59]* Diabetes 44 (32.8) [p 0.02]* Liver disease 26(19.4) [p 0.09]* COPD 42 (31.3) [p 0.21]* *Severe persistent lymphopenia* Age 65.3 ± 12.5 Female 25(32.9) APACHE II 18.6 (5.8) Congestive heart failure 26 (34.2) Diabetes 30 (39.5) Liver disease 18 (23.7) COPD 33 (43.4)	Day 4 ALC (4 days after blood culture taken)	Nosocomial infection = culture positive infections identified >48 hours after primary bacteraemia and arising from secondary source Moderate lymphopenia 27 (20.1) OR 1.60 (0.83, 3.11) *[p 0.16]* Severe persistent lymphopenia 19 (25) OR 2.11 (1.02, 4.39) *[p 0.04]*	*28-day mortality* 63 (11.0) *1-year mortality* 98 (46.7)	Hospital LOS 14.8 ±9.4 [p 0.37]* ICU LOS 4.4 ± 4.3 [p 0.15]*
**Imabayashi *et al.* 2022 ** [36] University hospital 2012 to 2016 R*	Consecutive patients who underwent spinal surgery. Exclusion of patients treated with unplanned antibiotics (except for prophylactic surgery) 329	≤1	61 (18.5)	Age 72 (15-85) (median, IQR) [ p 0.013]* Female 17 (27.9)	Post operative (day 7)	Surgical site infection (SSI), defined according to criteria of the Centres of Disease Control and Prevention. Diagnosis was made by attending surgeon based on the need for debridement, blood cultures that were positive for infectious agents or draining of surgical wound within 4 weeks 4 (6.56) [p 0.065]* OR 7.54 (1.91-29.83) [*p 0.004]* Univariate analysis on lymphopenia preoperatively on SSI OR 0 (0.001- 0.002) [*p 0.997]*	N/A	N/A
**Koch *et al.* 2022 ** [37] USA, trauma centre hospital May 2009 to December 2018 R*	Adults admitted with Chest Abbreviated Injury Scale (CAIS) ≥2. Exclusion of patients who died within 24 hours of admission, bowel perforation on admission, penetrating trauma and burns 1394	≤1	618 (44.3)	Age 57.4 ± 22.3 [p <0.001]* Female 185 (29.9) Heart failure 37 (6) [p 0.019]*	Within 24 hours of admission	Pneumonia 95 (15.4) [ p 0.317 ]* UTI 60 (9.7) [p 0.453 ]* Wound infection 13 (2.3) [ p 1.00]* Other infection 16 (2.6%) [ p 1.00 ]*	*In-hospital mortality* 60 (9.7)	Hospital LOS 9.7 ± 7.4 [p<0.001]* OR 1.151 (1.03-1.29] *[p 0.017]* ICU LOS 5.05 ± 5.20 [p 0.797]*
**Mendez *et al.* 2019 ** [38] Spain, tertiary hospital Not reported P**	≥18 years admitted with CAP Exclusion of nursing home patients, life expectancy less than 3 months, immunosuppression, and hospital admission for ≥48 hours in the preceding 15 days 217	≤0.724 (based on Bermejo-Martin et al.) Analysis also carried out on ALC ≤1	ALC ≤ 1.0 128 (59.0) ALC ≤ 0.724 85(39.2)	ALC ≤0. 724 Age 72.9 ± 15.1 [p<0.001]* Female 28 (32.9) Diabetes 19 (22.4) [p 0.749]* Heart disease 31 (36.5) [p 0.120]* Liver disease 3 (3.5) [p 0.839]* COPD 27 (31.8) [p 0.010]*	First morning after hospital admission	N/A	N/A	ALC ≤1.0 Hospital LOS 7 ± 2.0 [p 0.0040]* ALC ≤0.724 Hospital LOS 7 ± 3.0 [p 0.176]*
**Rubio-Rivas *et al*.** **2015** Spain 2012 to 2013 P**	All consecutive ≥ 75 years admitted for medical conditions 180	<1.1	45 (25)	Age 84.5 ± 5.0 [p 0.312] Female 25 (55.6)	Admission	*Infection as cause of admission 1 (2.2)*	In hospital mortality 12 (26.7) [p 0.001]* OR 3.9 *[p 0.03] CIs not reported* 1 year mortality multivariate analysis HR 1.9 [*p 0.038] CIs not reported*	Hospital LOS 19.9 ± 12.2 [p 0.002]*
**Ruiz *et al*. 2023 ** [43] Spain, 2 hospitals January 2002 to December 2020 P**	≥18 years admitted with pneumococcal CAP (based on the results of pneumococcal urinary antigen test and/or blood culture) 1173	<1 Severe lymphopenia: ALC below 0.5	686 (58.4) Severe lymphopenia 282 (24)	Mean age not provided Female 259 (37.8) Pneumonia Severity Index (PSI)>3 381 (55.5) [p<0.0001]*	Admission	N/A *Septic shock subgroup (based on ALC < 0.5)* 66 (23.4) *[p<0.001]*	In-hospital mortality (severe lymphopenia) 30 (10.6) *[p<0.001]*	N/A
**Vulliamy *et al*. 2015** UK, district general hospital January 2002 to October 2013 R*	Emergency general surgical patients admitted to ICU with acute intra-abdominal pathology and SOFA score≥5 at time of ICU admission 173	<1 Persistent lymphopenia: ALC below the lower limit of normal (defined as 1-3.0 x 10^9^/L) throughout the 7-day period or until death	61 (35.3) with persistent lymphopenia	Age 72.6 ± 15.2 [p<0.01]* APACHE II 19 (15-23) [p<0.01]*	ALC from ICU admission to day 7 of admission	N/A	In-hospital mortality 39 (64) [p<0.01}* OR 3.36 (1.60-7.04) *[p<0.01]*	N/A
**Wittermans *et al*. 2022 ** [41] The Netherlands, multicentre study Post hoc analysis of RCT	Non-ICU hospitalised CAP Exclusion of immunosuppressed patients 354	≤0.71	117 (33.1)	Age 68 ± 15 [p 0.002]* Female 38 (32.5) COPD 19 (16) [p 0.36]* Diabetes 29 (25) [p 0.021]* Congestive heart failure 15 (13) [ p 0.057]* Liver disease 2 (2) [p 0.47]*	ALC on Emergency Department presentation	N/A	30-day mortality 5 (4) [p = 0.37]*	Hospital LOS 5.5 ± 3.4 [p 0.61]*
**Zhou *et al*. 2018 ** [42] China, 424 hospitals September 2009 to December 2009 P**	RT-PCR positive for influenza A (H1N1) on admission Exclusion of secondary bacterial or fungal infection within 48 hrs of hospitalisation 2146	<0.8	821 (38.3)	N/A	N/A	Nosocomial infection: clinical symptoms/signs of pneumonia or bacteraemia and positive culture of a new pathogen ≥48 hours after admission 148 (65.5) [p<0.001]* OR 1.906 (1.361-2.671) *[p <0.001]*	N/A	N/A

Nine studies were retrospective observational cohort studies and six were prospective cohort studies (Table 1). Lymphopenia was variably defined in all included papers, with a range of 0.5 to 1.2 x 10^9^/L. Two studies did not report when ALC measures were collected [16, 42]. Eight studies reported ALC measures either on admission or within 24 hours of admission [17, 33, 34, 37–39, 41, 43]. Two studies measured ALC on specific days determined a priori; 4^th^ day after a blood culture was taken in septic patients and postoperative day 7, respectively [15, 36]. Three studies measured ALC at multiple time points during hospital admission [32, 35, 40].

### Risk of bias

**Fig. 2:**
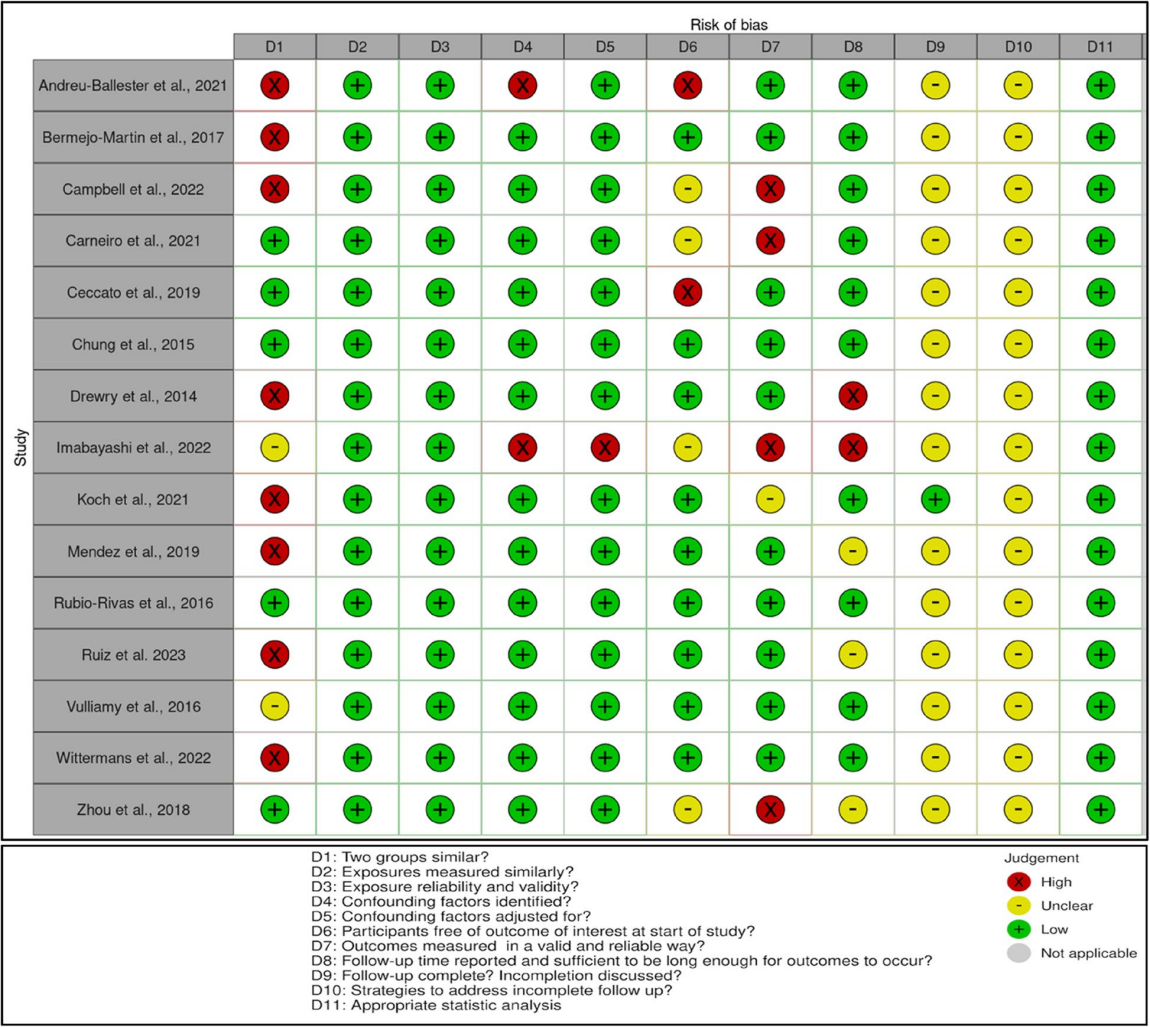
Risk of bias using the JBI Critical Appraisal Checklist for Cohort Studies [26]. Plot created using *robvis* software [44]

Risk of bias was high amongst most included studies (Fig. 2). Areas of high risk of bias or uncertainty related to three main issues. Firstly, there were significant baseline differences between lymphopenic and non-lymphopenic groups in measures of demographics and disease severity. Secondly, completeness of data or strategies to deal with missing data were not reported. Thirdly, it was often unclear whether participants were free of the outcome at start of the study as these were not always measured in a reliable way.

Funnel plots were visually inspected for identification of publication bias, where more than five publications reported a specific outcome. Visual inspection of funnel plots demonstrated high risk of publication bias.

### Pooled prevalence of lymphopenia

**Fig. 3:**
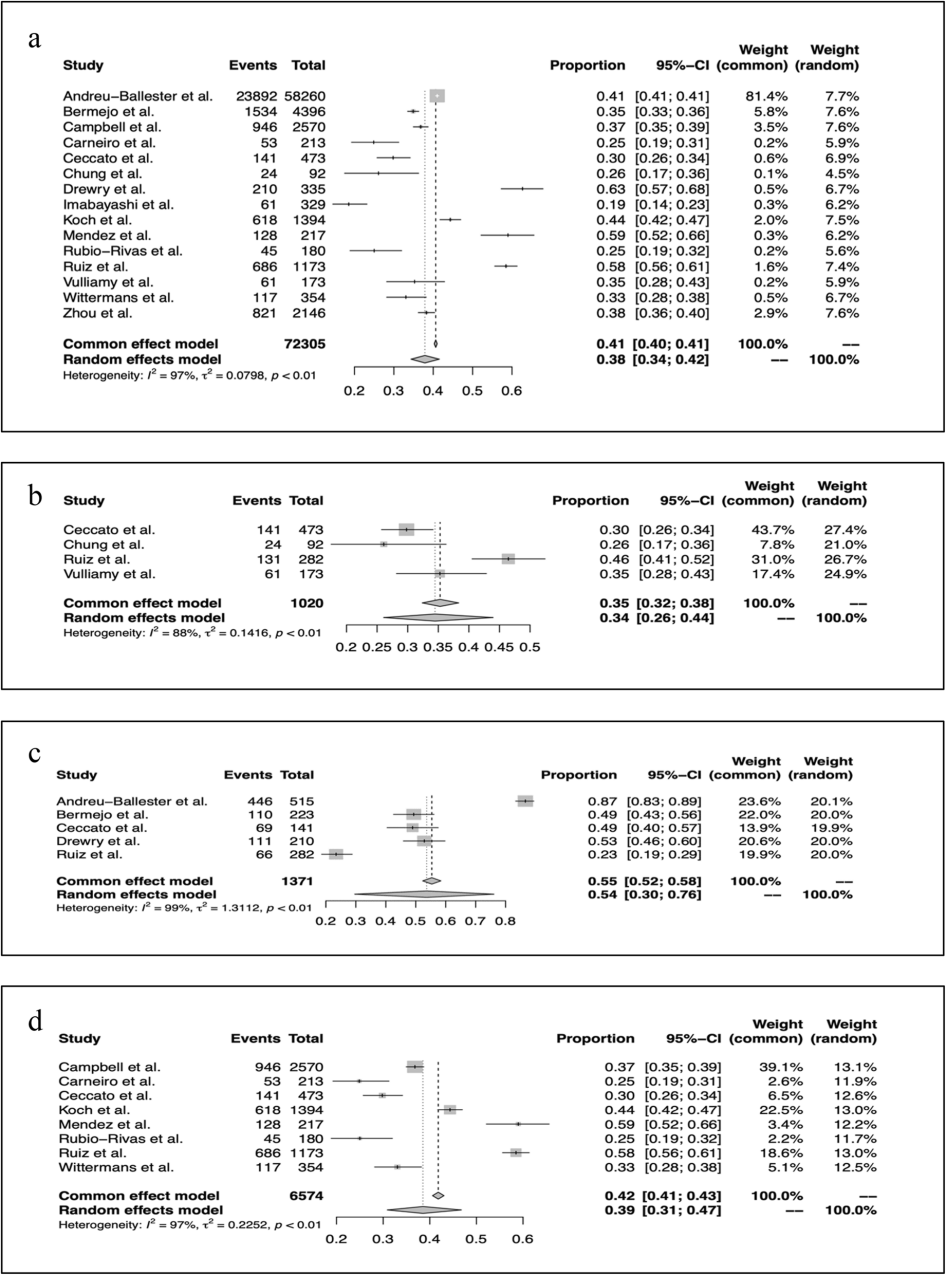
**a** Forest plot of pooled prevalence of lymphopenia (of any definition) across 15 studies. CI, confidence interval. **b** Subgroup analysis of ICU pooled prevalence of lymphopenia of any definition*. ***c** Subgroup analysis of septic shock pooled prevalence of lymphopenia of any definition. **d** Subgrouping of lymphopenia based on absolute lymphocyte count at time of admission (ALC, x 10^9^/L)

The pooled prevalence of lymphopenia in all-cause hospitalisations was 38% (random effects model proportion 0.38; CI 0.34-0.42, I^2^ = 97%, *p< 0.01)* (Fig. 3a).

Given the significant study-related heterogeneity across all included studies, further subgroup analysis was carried out. Studies investigating an ICU population (*n* = 1020) demonstrated lymphopenia in 34% of admissions (random effects model proportion 0.34; 95% CI 0.26-0.44, I^2^ = 88%, *p< 0.01*) (Fig. 3b), although heterogeneity remained high [34, 35, 40, 43]. Subgroup analysis for septic shock populations (*n* = 1371) demonstrated higher prevalence of 54% with significant heterogeneity (random effects model proportion 0.54; 95% CI 0.30-0.76, I^2^ = 99%, *p< 0.01*) (Fig. 3c) [15, 16, 32, 34, 43].

Lymphopenia definition varied across studies, however eight studies defined lymphopenia based on ALC at hospital admission and/or within 24 hours of admission [17, 33, 34, 37–39, 41, 43]. Admission lymphopenia had a prevalence of 39% (random effects model proportion 0.39; 95% CI 0.31-0.47, I^2^ = 97%, *p< 0.01*) (Fig. 3d). Heterogeneity remained high.

### Lymphopenia and infection

**Fig. 4:**
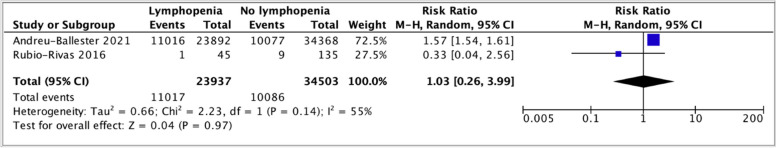
Forest plot of infection (as cause of admission) and lymphopenia (of any definition). CI, confidence interval; M-H, Mantel-Haenszel

Two studies reported infection as the cause of admission [32, 39]. Lymphopenia was not associated with an infection diagnosis (RR 1.03; 95% CI 0.26-3.99, *p=*0.97, I^2^ = 55%) (Fig. 4). Subgroup analysis for this outcome was not possible due to the small number of studies. However, heterogeneity is noted between studies by Andreu-Ballester *et al*. and Rubio-Rivas *et al*.; lymphopenia definition (< 1 *vs* < 1.1 x 10^9^/L), timing of lymphocyte measures (any point during hospital admission *vs* admission), and study population characteristics including age (> 14 *vs* ≥ 75 years), respectively (Table 1) [32, 39].

**Fig. 5:**
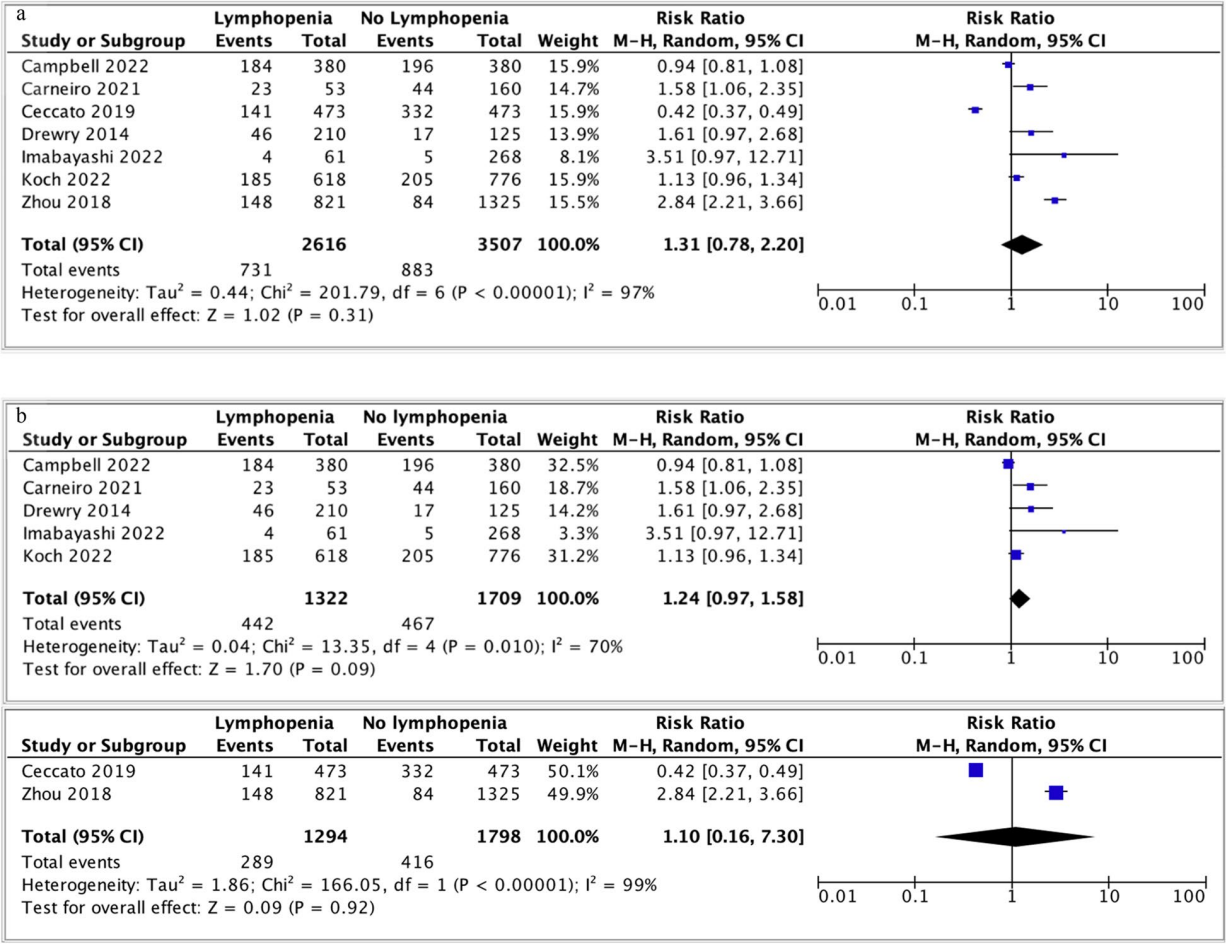
**a** Forest plot of nosocomial infection and lymphopenia (of any definition). Funnel plot analysis demonstrates asymmetric shape. **b** Forest plot of nosocomial infection and lymphopenia, stratified by lymphopenia definition. Top panel ALC < 1.2 > 0.8 x 10^9^L *vs* bottom panel ALC < 0.8 x 10^9^L. CI, confidence interval; M-H, Mantel-Haenszel

Seven studies reported the outcome of healthcare-associated infection and lymphopenia [15, 17, 33, 34, 36, 37, 42]. Lymphopenia was not associated with healthcare-associated infection (RR 1.31; 95% CI 0.78-2.20, *p*=0.31, I^2^ = 97%) (Fig. 5a). Sensitivity analysis was carried out based on lymphopenia definition, stratified as either less than 1.2 or less than 0.8 x 10^9^/L (Fig. 5b). Heterogeneity was reduced in the analysis of ALC less than 1.2 x10^9^/L but increased for studies of ALC greater than 0.8 x 10^9^/L. The outcome remained non-significant.

**Fig. 6:**
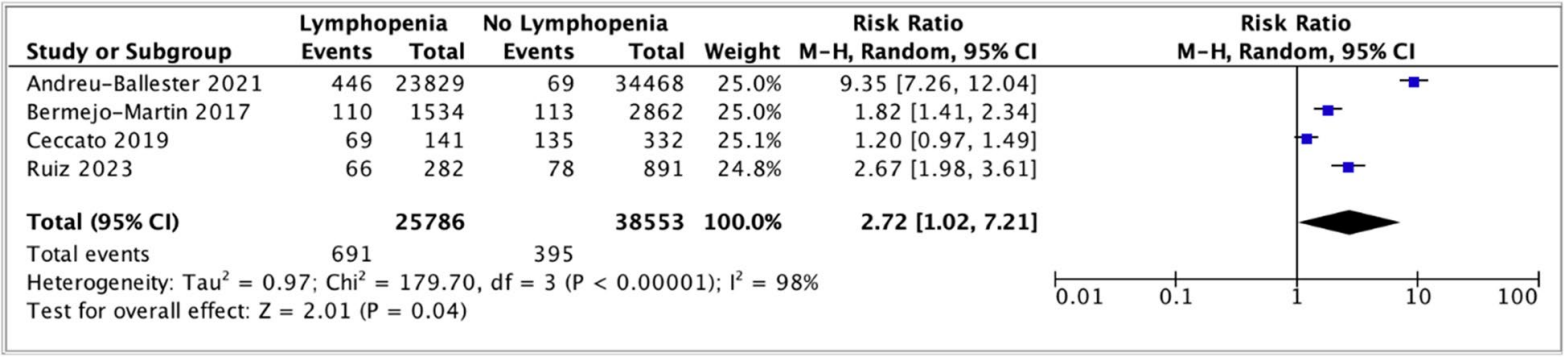
Forest plot of septic shock and lymphopenia (ALC stratified by cut-off, x 10^9^/L). CI, confidence interval; M-H, Mantel-Haenszel

Four studies reported the outcome of septic shock and lymphopenia [16, 32, 34, 43]. Lymphopenia was associated with septic shock (RR 2.72; 95% CI 1.02–7.21, *p* = 0.04, I^2^ = 98%) (Fig. 6). Heterogeneity was high between the studies.

### Lymphopenia and mortality

**Fig. 7:**
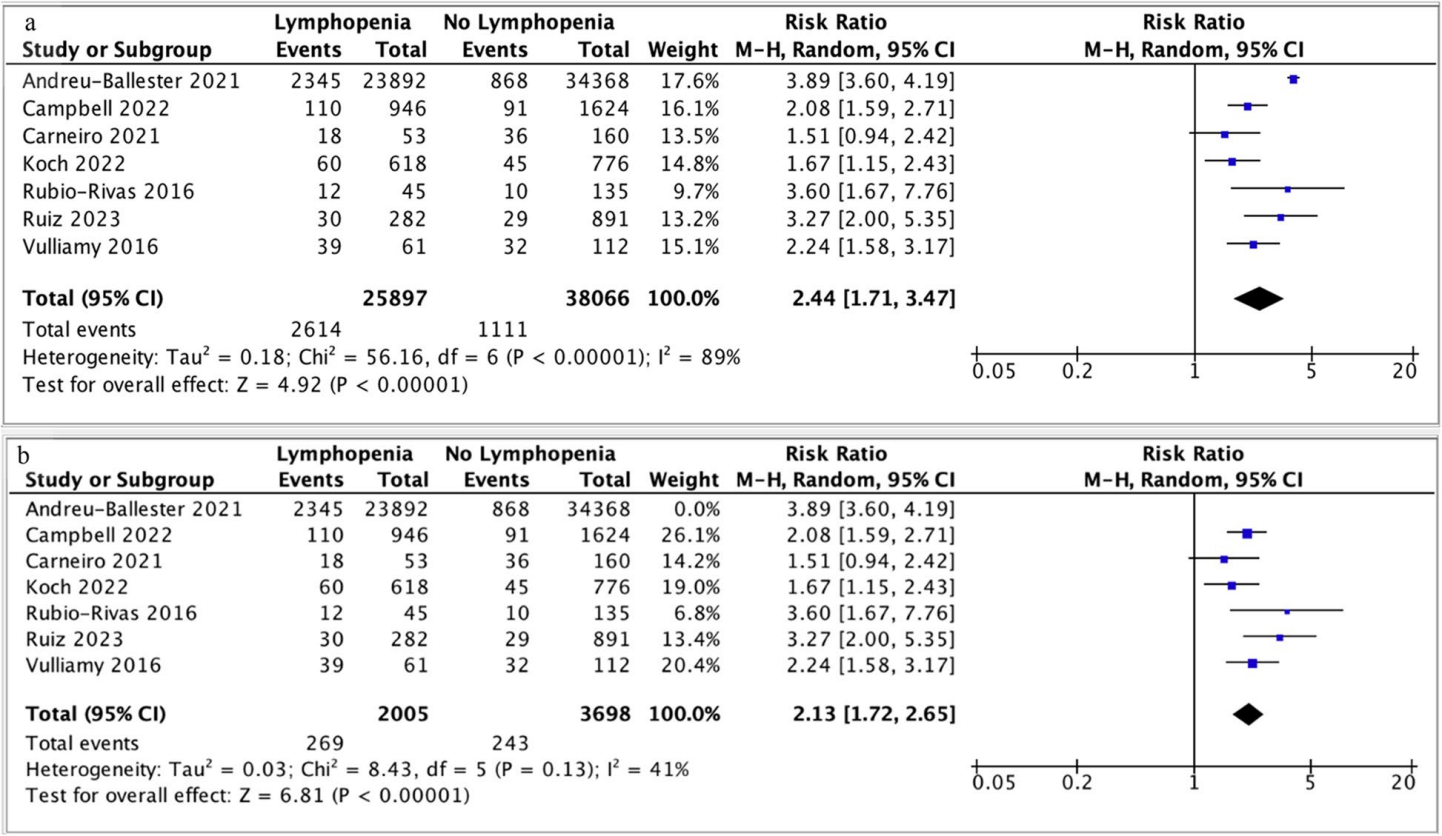
**a** Forest plot of in-hospital mortality and lymphopenia (ALC stratified by 1.1 x 10^9^/L as cut-off). Funnel plot demonstrates asymmetry. **b** Exclusion of Andreu-Ballester *et al*. changes to I^2^ = 41%. CI, confidence interval; M-H, Mantel-Haenszel

In-hospital mortality was reported by seven studies (Fig. 7) [17, 32, 33, 37, 39, 40, 43]. Lymphopenia was associated with higher in-hospital mortality (RR 2.44; 95% CI 1.71-3.47, p < 0.00001, I^2^ =89%) (Fig. 7). Excluding Andreu-Ballester *et al*.’s study, which analysed data from 58260 hospital admissions, the significant heterogeneity is reduced to 41% (RR 2.13, 95% CI 1.72-2.65, p < 0.00001, I^2^ =41%) (Fig. 7b) [32].

**Fig. 8:**
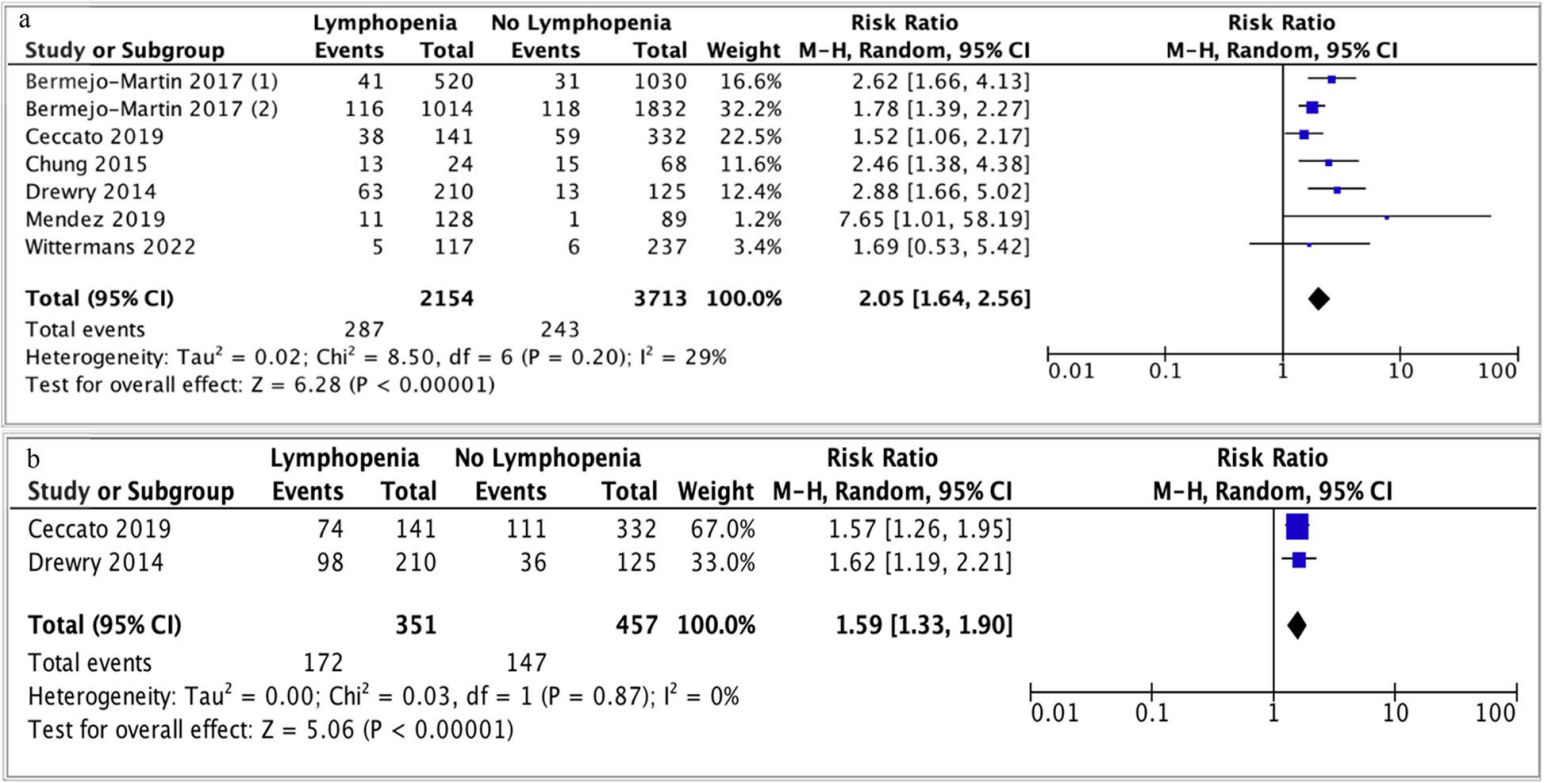
**a** Forest plot of 28/30-day mortality (early) with lymphopenia of any definition. Bermejo-Martin *et al*. (1) and (2): data from derivation cohort and verification cohort, respectively. Funnel plot demonstrates asymmetry. **b** Forest plot of 90-day/1-year mortality (late) with lymphopenia of any definition. CI, confidence interval; M-H, Mantel-Haenszel

‘Early’ (28/30-day) mortality was reported in six studies [15, 16, 34, 35, 38, 41]. Bermejo-Martin *et al*. studied two cohorts; ‘derivation’ multisite and ‘validation’ single-site cohorts annotated as [1] and [2], respectively (Fig. 8a) [16]. Lymphopenia was associated with higher early mortality (RR 2.05; 95% CI 1.64-2.56, *p <* 0.00001, I^2^ = 29%) (Fig. 8a). ‘Late’ (90-day/1-year) mortality was reported in two studies [15, 34]. Lymphopenia was associated with higher late mortality (RR 1.59; 1.33-1.90, *p <* 0.00001, I^2^ = 0%) (Fig. 8b).

### Lymphopenia and length of stay

**Fig. 9:**
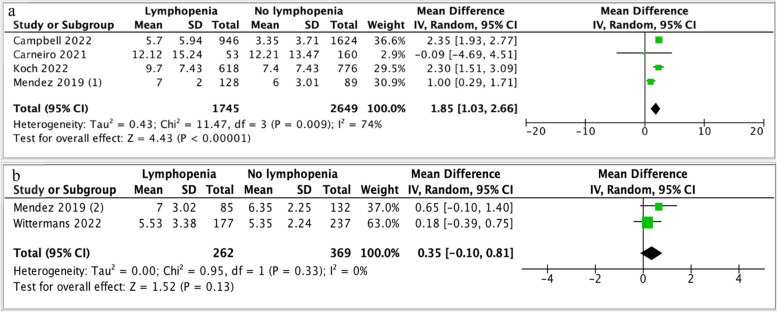
Forest plot of hospital Length of Stay (LOS) and lymphopenia. Mendez *et al*. (1) and (2): absolute lymphocyte count cut off less than 1.0 x10^9^/L *vs*. less than 0.724 x10^9^/L, respectively. **a** Lymphopenia defined ALC less than 1 x 10^9^/L. **b** Lymphopenia defined as ALC < 0.724 x10^9^/L. CI, confidence interval; I-V, Inverse Variance method

Hospital length of stay (LOS) was reported in five studies (Fig. 9) [17, 33, 37, 38, 41]. Mendez *et al.* reported hospital LOS of a population of patients with CAP based on two lymphopenia definitions of ALC ≤ 1 x10^9^/L and ALC ≤ 0.724 x 10^9^/L [38]. LOS has been reported separately in the analysis for these two lymphopenia thresholds (Fig. 9) [38]. The overall mean difference in hospital LOS is 1.25 days (95% CI 0.32-2.18, *p =* 0.008, I^2^ = 89%) longer for patients with lymphopenia (Fig. 7).

Sensitivity analysis was performed based on lymphopenia definition. Four studies, defining lymphopenia as less than 1 x 10^9^/L, demonstrated that lymphopenic populations stayed in hospital 1.85 days (95% CI 1.03-2.66, *p <* 0.0001, I^2^ = 74%) longer than non-lymphopenic populations [17, 33, 37, 38]. Two studies, defining lymphopenia as less than 0.724 x 10^9^/L, demonstrated that lymphopenic patients stayed in hospital for 0.35 days longer than the non-lymphopenic population, however this was not statistically significant (95% CI -0.10-0.81, *p =* 0.13, I^2^ = 0%) [38, 41].

**Fig. 10:**
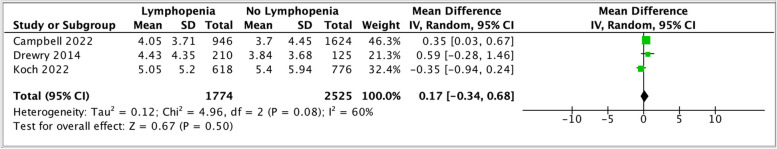
Forest plot of ICU LOS and lymphopenia (of any definition). CI, confidence interval; I-V, Inverse Variance method

ICU LOS was reported by three studies (Fig. 10) [15, 33, 37]. Mean difference was 0.17 days longer for non-lymphopenic subgroup, however this was not statistically significant (95% CI -0.34-0.68, *p=* 0.50, I^2^ = 60%) (Fig. 10).

## Discussion

This systematic review adds to the growing evidence that lymphopenia is associated with adverse clinical outcomes. We demonstrate that lymphopenia is common in hospitalised patients, occurring in 38% of patients. We demonstrate that lymphopenia is associated with increased early and late mortality. In addition, there is prolonged hospital stay. The analysis did not demonstrate a significant difference in risk of admission with an infection or acquiring a hospital-acquired infection if lymphopenic. However, there was an increased risk of septic shock in lymphopenic patients. The fifteen studies included demonstrate that lymphopenia is seen across a wide range of pathologies including infection, trauma, and intracranial haemorrhagic conditions.

Lymphopenia has been associated with increased mortality and infection risk in a wide range of settings including community populations, perioperative, and sepsis [15, 18, 19, 45]. The studies have broadly shown that lymphopenia is associated with an increased risk of infections and mortality. Given the range of clinical settings in which lymphopenia has been shown to result in adverse clinical outcomes, we summarised for the first time, the prevalence of lymphopenia in all-cause hospitalised patients. Our findings are broadly in line with other studies. In a meta-analysis of peri-operative patients, lymphopenia was associated with a three-fold increase in mortality and a higher rate of postoperative complications and infections [45]. While we demonstrated increased mortality in both ‘early’ and ‘late’ deaths, we did not demonstrate an increase in risk of infection. An increased risk of infection seems intuitively associated with lymphopenia. Lymphopenia is a hallmark of immune dysfunction in sepsis and is associated with healthcare-associated infections [46]. In a single centre observational study, it was persistent lymphopenia lasting beyond the fourth day of sepsis admission, that was associated with a significant increase in secondary infections [15]. Furthermore, in a large population study of 98, 344 individuals, lymphopenia was associated with an increased risk of acquiring infections, including sepsis [18]. When summarising the risk of infection across a broad range of conditions, we did not find a significant association between an infectious cause of hospital admission or healthcare-associated infection. We did, however, show a 3-fold increased risk of septic shock with lymphopenia (RR 2.72; 95% CI 1.02–7.21, *p* = 0.04, I^2^ = 98%).

Our review suggests there is a ‘dose-response’ between severity of lymphopenia and adverse clinical outcome. In a retrospective study, Bermejo-Martin *et al.,* identified a subgroup of patients with CAP who were lymphopenic (ALC less than 0.724 x 10^9^/L) that accounted for a significant portion of individuals who developed septic shock and demonstrated a significantly higher risk of 30-day mortality [16]. Consistent with this finding, in a large cohort study, Andreu-Ballester *et al*. demonstrated that the lowest absolute values were demonstrated in sepsis and septic shock, with severe low absolute counts of lymphocytes associated with higher risk of mortality [32]. Drewry *et al*. stratified lymphopenia definitions as moderate and severe persistent lymphopenia. This stratification demonstrated a higher incidence of nosocomial infections alongside higher 28-day and 1-year mortality rates in the severe cohort compared to the moderate cohort [15]. These findings suggest a relationship between severity of lymphopenia and outcome, specifically in subgroups of septic shock.

Given the spectrum of conditions that lymphopenia is present in, there is uncertainty whether lymphopenia is an epiphenomenon of an unwell patient or whether it plays a central role in morbidity and mortality. The significance of lymphopenia in different clinical settings and populations is uncertain. Studies to date indicate that lymphopenia reflects a wider dysfunctional immune system. This is certainly shown in studies in sepsis, where immune dysfunction is characterised not only by lymphopenia, but also low monocyte HLA-DR, increased PD-1 and increased regulatory T cells [5–9]. Other routinely measured biomarkers reflect immune dysfunction and have been shown to be associated with increased mortality. In a large population of 31,178 outpatients, in addition to lymphopenia, high levels of C-reactive protein (CRP) were also associated with reduced survival [19]. A follow-up study of sepsis survivors identified a hyperinflammation/immunosuppression phenotype with a significantly higher 1-year mortality risk, demonstrated by CRP as a marker of ongoing inflammation and additional markers of immunosuppression including soluble PD-L1 [10]. Although our review cannot conclude that lymphopenia in the included studies is due to immune dysfunction, our findings are consistent with current understanding of immune perturbations in acute illness.

There are limitations to this study. We aimed to determine the prevalence of lymphopenia in a ‘general’ hospital population. For this reason, we excluded studies that specifically focused on immunosuppressed populations in which the prevalence of lymphopenia and the associated infection risk would be much higher. These populations are immunosuppressed secondary to medical treatments for cancer or inflammatory diseases, and so represent a different population to those with immune dysfunction because of an acute disease. However, it is possible that some patients within the included studies of ‘general’ populations would be on immunosuppressive medications and contribute to the lymphopenic population. In addition, it can be argued that patients with COVID-19 should be represented in the general in-patient population. We excluded these studies because lymphopenia is a well-recognised characteristic and systematic review of lymphopenia in COVID-19 patients has been recently published [23].

This review is further limited by the range of lymphopenia definitions used in the studies, resulting in high levels of heterogeneity in the meta-analysis. Definition of lymphopenia ranged from 0.5 to 1.2 x 10^9^/L. The lack of a unified definition of lymphopenia demonstrates the need for further research in causality, and in clarifying whether there is a potential count-dependent relationship between severity of lymphopenia and outcome.

**Fig. 11:**
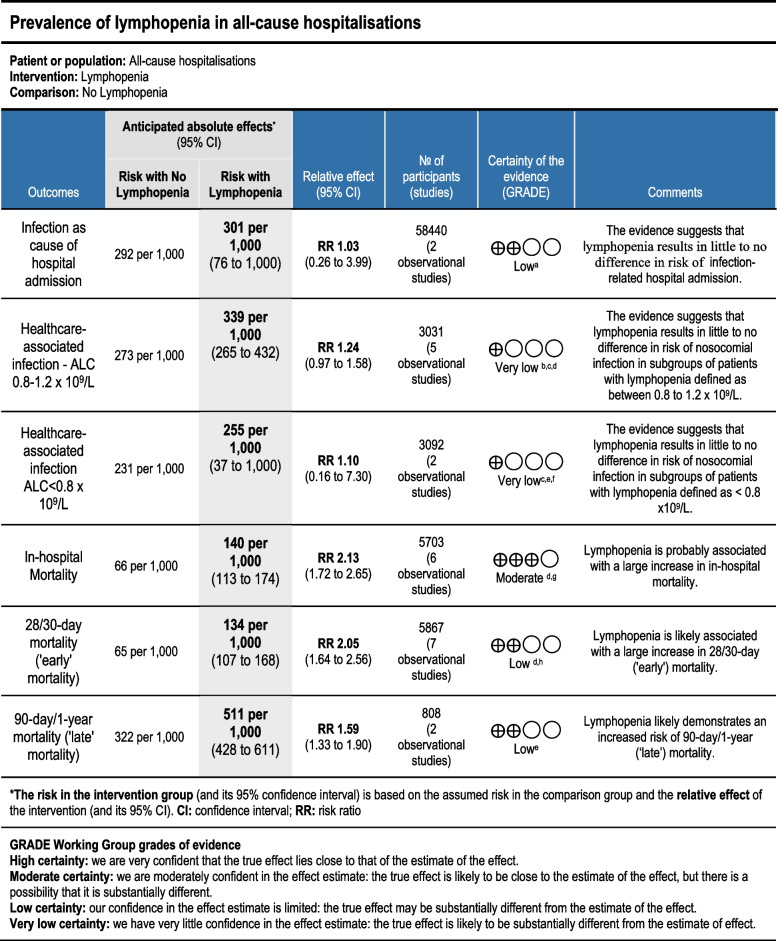
Summary of findings table and GRADE assessment of outcomes. **a** Wide confidence intervals for overall effect estimate. **b** Lack of confounding variables identification [17, 33, 36]. Follow-up time not reported/unclear [15, 36]. Significant baseline differences between lymphopenic and non-lymphopenic subpopulations [37]. **c** Statistically significant high heterogeneity. **d** Asymmetrical funnel plot. **e** Ceccato *et al*. determined lymphopenia cut-off based on previous analysis [34]. **f** Lack of generalisability to wider hospitalised population; Ceccato *et al*.'s study population was related to intensive care-related nosocomial infection while Zhou *et al.*'s study concentrated on patients with severe influenza A patients [34, 42]. **g** Significant differences between lymphopenic and non-lymphopenic subpopulations [37, 43]. Unclear reporting of characteristics across groups in Andreu-Ballester *et al*. and Vulliamy *et al*. [32, 40] **h** Significant differences between lymphopenic and non-lymphopenic subpopulations [16, 38, 41]. Potential confounding factors not identified [34]

Lastly, the conclusions made by this meta-analysis are limited by the quality of studies included. Most of the studies had a high risk of bias or uncertainty regarding risk of bias. Since the included studies were observational studies, the GRADE quality of evidence was often downgraded (Fig. 11). However, large sample sizes in studies such as Andreu-Ballester *et al.*, Bermejo-Martin *et al.,* and Campbell *et al.* allowed upgrading of quality due to large effect size demonstrated across multiple outcomes (Fig. 11) [16, 32, 33].

In conclusion, this meta-analysis shows that lymphopenia is common across all-cause hospitalisations and associated with increased risk of mortality and length of stay. Moreover, given the consistent findings across several types of pathology, the data suggest a link between lymphopenia at any point during a hospital stay and poor outcome. This meta-analysis highlights the paucity of available high-quality evidence. By summarising the prevalence of lymphopenia in hospitalised patients, this review may inform the design of future studies investigating outcomes and novel treatments for immune dysfunction in hospitalised patients. In particular, prospective studies of lymphocyte count and its potential correlation with detailed immunophenotyping and longer term patient outcomes may provide further insight into the value of lymphopenia as a marker of immune dysfunction and prediction of illness trajectory after hospitalisation.

### Supplementary Information

**Additional file 1: Supplementary Table 1.** The pre-specified imputation algorithm for the seven-category ordinal scale.

## Data Availability

The datasets generated and analysed during the current study are available below. Embase search strategy: https://ovidsp.ovid.com/ovidweb.cgi?T=JS&NEWS=N&PAGE=main&SHAREDSEARCHID=68ESh1P8ZoHE1kmkNz5h48BVP89Yt1WPJaCI8ngQgah8cAWJcroVMyVvC1b3XNmtQ MEDLINE search strategy: https://ovidsp.ovid.com/ovidweb.cgi?T=JS&NEWS=N&PAGE=main&SHAREDSEARCHID=7Cj8rfrUOTD7CIIlkeLwDOPZ5ZiSJ1mGva3uMLQ3fCkAiPYdXDsB5ss6FZ0Glvvs2 CENTRAL search strategy: *"lymphopenia" OR lympho?enia OR lymphocyte** *AND* *hospital* OR "critical care" OR "intensive care"* *AND* *infection** *AND* *"randomi*ed controlled" OR "randomi*ed clinical" OR "randomi*ed intervention" OR "controlled clinical" OR observational*

## Abbreviations

(PRISMA) guidelines: Preferred Reporting Items for Systematic Reviews and Meta-Analysis
(SOFA) score: Sequential organ failure assessment
(APACHE II) score: Acute physiology and chronic health evaluation II
SD: Standard deviation
ICU: Intensive care unit
ALC: Absolute lymphocyte count
(JBI) critical appraisal checklist for observational studies: Joanna Briggs Institute
(GRADE) assessment: Grading of Recommendations, Assessment, Development and Evaluations
RR: Risk ratio
OR: Odds ratio
CIs: Confidence intervals
(I-V) method: Inverse variance
(M-H) method: Mantel-Haenszel
MD: Mean difference
CAP: Community-acquired pneumonia
VAP: Ventilator-associated pneumonia
COPD: Chronic obstructive pulmonary disease
LOS: Length of stay
