# Supplementary material for: Pooled prevalence of lymphopenia in all-cause hospitalisations and association with infection: a systematic review and meta-analysis
Source: BMC Infect Dis. 2023 Dec 2;23:848. doi: 10.1186/s12879-023-08845-1 (PMC10693046; doi:10.1186/s12879-023-08845-1)
Supplement: Supplementary file 1 — Additional file 1: Supplementary Table 1. The pre-specified imputation algorithm for the seven-category ordinal scale. [file 12879_2023_8845_MOESM1_ESM.docx]

**Prevalence of lymphopenia/ absolute lymphocyte count not reported (n = 103)**

1. Akatsuka M, Tatsumi H, Sonoda T, Masuda Y. Low immunoglobulin G level is associated with poor outcomes in patients with sepsis and septic shock. Journal of Microbiology, Immunology and Infection. 2021;54:728-32.
2. Akilli NB, Yortanli M, Mutlu H, Gunaydin YK, Koylu R, Akca HS, et al. Prognostic importance of neutrophil-lymphocyte ratio in critically ill patients: Short- and long-term outcomes. American Journal of Emergency Medicine. 2014;32:1476-80.
3. Alkhamis T, Ivic D, Wagner J, Ivic J, Dobrosevic B, Turina I, et al. Postoperative immunosuppression markers and the occurrence of sepsis in patients with benign and malignant disease. Wiener Klinische Wochenschrift. 2014;126:774-84.
4. Brakenridge SC, Efron PA, Stortz JA, Ozrazgat-Baslanti T, Ghita G, Wang Z, et al. The impact of age on the innate immune response and outcomes after severe sepsis/septic shock in trauma and surgical intensive care unit patients. Journal of Trauma and Acute Care Surgery. 2018;85:247-55.
5. Dos Santos Junqueira JC, Cotrim Soares E, Rodrigues Correa Filho H, Fenalti Hoehr N, Oliveira Magro D, Ueno M. Nutritional risk factors for postoperative complications in Brazilian elderly patients undergoing major elective surgery. Nutrition. 2003;19:321-6.
6. Feng Y, Wu Q, Zhang T, Chen J, Wu X. Natural killer cell deficiency experiences higher risk of sepsis after critical intracerebral hemorrhage. International Journal of Immunopathology and Pharmacology. 2021;35.
7. Goldberg I, Shalmon D, Shteinvil R, Wasserman A, Berliner S, Levinson T, et al. The superiority of 72 h leukocyte descent over CRP for mortality prediction in patients with sepsis. Clinica Chimica Acta. 2020;514:34-9.
8. Guo L, Wei D, Zhang X, Wu Y, Li Q, Zhou M, et al. Clinical Features Predicting Mortality Risk in Patients With Viral Pneumonia: The MuLBSTA Score. Frontiers in Microbiology. 2019;10.
9. Guo M, Tong Z. Risk factors associated with invasive pulmonary mycosis among severe influenza patients in beijing city, china. International Journal of General Medicine. 2021;14:7381-90.
10. He L, Wang J, Wang F, Zhang L, Zhang L, Zhao W. Increased neutrophil-to-lymphocyte ratio predicts the development of post-stroke infections in patients with acute ischemic stroke. BMC Neurology. 2020;20(1):328.
11. He Q, Tang W, Deng Y, He Y, Xie L, Qin X, et al. The diagnostic value of neutrophil-to-lymphocyte ratio and platelet-to-lymphocyte ratio in tuberculous spondylitis. International Journal of Clinical and Experimental Medicine. 2016;9:16360-6.
12. Hohlstein P, Gussen H, Bartneck M, Warzecha KT, Roderburg C, Buendgens L, et al. Prognostic relevance of altered lymphocyte subpopulations in critical illness and sepsis. Journal of Clinical Medicine. 2019;8.
13. Hou D, Wang C, Ye X, Zhong P, Wu D. Persistent inflammation worsens short-term outcomes in massive stroke patients. BMC Neurology. 2021;21.
14. Inose H, Kobayashi Y, Yuasa M, Hirai T, Yoshii T, Okawa A. Postoperative lymphocyte percentage and neutrophil-lymphocyte ratio are useful markers for the early prediction of surgical site infection in spinal decompression surgery. Journal of Orthopaedic Surgery. 2020;28(2).
15. Inose H, Okawa A. Predictors of surgical site infection after spinal instrumentation surgery: A consecutive series. European Spine Journal. 2019;28:2922.
16. Inoue S, Suzuki-Utsunomiya K, Okada Y, Taira T, Iida Y, Miura N, et al. Reduction of immunocompetent T cells followed by prolonged lymphopenia in severe sepsis in the elderly. Critical Care Medicine. 2013;41:810-9.
17. Irawany V, Lolobali MC. Neutrophil-to-lymphocyte Ratio as a New Predictor of Sepsis in Critically-ill Stroke Patients. Open Access Macedonian Journal of Medical Sciences. 2022:1118-21.
18. Iwata E, Shigematsu H, Koizumi M, Nakajima H, Okuda A, Morimoto Y, et al. Lymphocyte count at 4 days postoperatively and CRP level at 7 days postoperatively. Spine. 2016;41:1173-8.
19. Iwata E, Shigematsu H, Yamamoto Y, Ikejiri M, Okuda A, Sada T, et al. Temporal Evolution of White Blood Cell Count and Differential: Reliable and Early Detection Markers for Surgical Site Infection following Spinal Posterior Decompression Surgery. Spine Surgery and Related Research. 2022;6:271-8.
20. Jogia T, Lubstorf T, Jacobson E, Scriven E, Atresh S, Nguyen QH, et al. Prognostic value of early leukocyte fluctuations for recovery from traumatic spinal cord injury. Clinical and Translational Medicine. 2021;11.
21. Julien TP, Chan PH, Prentice HA, Paxton EW, Dillon MT, Navarro RA. Preoperative total lymphocyte count was not associated with adverse postoperative events following elective shoulder arthroplasty. Seminars in Arthroplasty JSES. 2021;31:239-47.
22. Kalkan ME, Kalkan AK, Gundes A, Yanartas M, Ozturk S, Gurbuz AS, et al. Neutrophil to lymphocyte ratio: a novel marker for predicting hospital mortality of patients with acute type A aortic dissection. Perfusion. 2017;32(4):321-7.
23. Kaya Y, Tas N, Canakci E, Cebeci Z, Ozbilen M, Keskin H, et al. Relationship of neutrophil-to-lymphocyte ratio with presence and severity of pneumonia. Journal of Clinical and Analytical Medicine. 2018;9:452-7.
24. Khan A, Riaz M, Kelly ME, Khan W, Waldron R, Barry K, et al. Prospective validation of neutrophil-to-lymphocyte ratio as a diagnostic and management adjunct in acute appendicitis. Irish Journal of Medical Science. 2017;187:379-84.
25. Khoury T, Daher S, Massarwa M, Hakimian D, Benson AA, Viener E, et al. A Validated Score Assessing the Risk of an Intra-Abdominal Abscess in Patients with Crohn's Disease Presenting at the Emergency Department. Journal of Crohn's and Colitis. 2019;13:1131-7.
26. Kim JE, Kim UJ, Kim HK, Cho SK, An JH, Kang SJ, et al. Predictors of viral pneumonia in patients with community-acquired pneumonia. PLoS ONE. 2014;9.
27. Kriplani A, it S, Chawla A, de la Rosette JJMCH, Laguna P, Jayadeva Reddy S, et al. Neutrophil-lymphocyte ratio (NLR), platelet-lymphocyte ratio (PLR) and lymphocyte-monocyte ratio (LMR) in predicting systemic inflammatory response syndrome (SIRS) and sepsis after percutaneous nephrolithotomy (PNL). Urolithiasis. 2022;50:341-8.
28. Krishnan M, Thodis E, Ikonomopoulos D, Vidgen E, Chu M, Bargman JM, et al. Predictors of outcome following bacterial peritonitis in peritoneal dialysis. Peritoneal Dialysis International. 2002;22:573-81.
29. Kudo D, Miyakoshi N, Hongo M, Kasukawa Y, Ishikawa Y, Mizutani T, et al. Relationship between preoperative serum rapid turnover proteins and early-stage surgical wound infection after spine surgery. European Spine Journal. 2016;26:3156-61.
30. Lan Y, Sun W, Chen Y, Miao J, Li G, Qiu X, et al. Nomogram Including Neutrophil-to-Lymphocyte Ratio for the Prediction of Stroke-Associated Infections. Frontiers in Neurology. 2020;11.
31. Laurent M, Bastuji-Garin S, Plonquet A, Bories PN, Le Thuaut A, Audureau E, et al. Interrelations of immunological parameters, nutrition, and healthcare-associated infections: Prospective study in elderly in-patients. Clinical Nutrition. 2014;34:79-85.
32. Lee H, Kim I, Kang BH, Um SJ. Prognostic value of serial neutrophil-to-lymphocyte ratio measurements in hospitalized community-acquired pneumonia. PLoS ONE. 2021;16.
33. Levochkina M, McQuillan L, Awan N, Barton D, Maczuzak J, Bianchine C, et al. Neutrophil-to-lymphocyte ratios and infections after traumatic brain injury: Associations with hospital resource utilization and long-term outcome. Journal of Clinical Medicine. 2021;10.
34. Li X, Li R, Cui J, He S, Zhang Q, Wang C. Predictive value of IL-6, PCT, NLR, and CRP in differentiating gram-negative bacterial bloodstream infections from gram-positive bacterial and fungal bloodstream infections in febrile patients. International Journal of Clinical and Experimental Medicine. 2019;12:7337-46.
35. Li Y, Ma YL, Gao YY, Wang DD, Chen Q. Analysis of the risk factors of postoperative cardiopulmonary complications and ability to predicate the risk in patients after lung cancer surgery. Journal of Thoracic Disease. 2017;9:1565-73.
36. Liang P, Yu F. Predictive Value of Procalcitonin and Neutrophilto-Lymphocyte Ratio Variations for Bloodstream Infection with Septic Shock. Medical Science Monitor. 2022;28.
37. Liesz A, Ruger H, Purrucker J, Zorn M, Dalpke A, Mohlenbruch M, et al. Stress Mediators and Immune Dysfunction in Patients with Acute Cerebrovascular Diseases. PLoS ONE. 2013;8.
38. Liu S, Li Y, She F, Zhao X, Yao Y. Predictive value of immune cell counts and neutrophil-to-lymphocyte ratio for 28-day mortality in patients with sepsis caused by intra-abdominal infection. Burns and Trauma. 2021;9.
39. Liu S, Wang X, She F, Zhang W, Liu H, Zhao X. Effects of Neutrophil-to-Lymphocyte Ratio Combined With Interleukin-6 in Predicting 28-Day Mortality in Patients With Sepsis. Frontiers in Immunology. 2021;12.
40. Liu X, Shen Y, Wang H, Ge Q, Fei A, Pan S. Prognostic Significance of Neutrophil-to-Lymphocyte Ratio in Patients with Sepsis: A Prospective Observational Study. Mediators of Inflammation. 2016;2016.
41. Lopez-Mestanza C, Andaluz-Ojeda D, Gomez-Lopez JR, Bermejo-Martin JF. Lymphopenic hospital acquired sepsis (L-HAS): An immunological phenotype conferring higher risk of mortality. Medicina Intensiva. 2019;43:510-2.
42. Lu J, Liu H, Cao LL, Zheng CH, Li P, Xie JW, et al. The Granulocyte-to-Lymphocyte Ratio as a Marker of Surgical Stress and a Predictor of Postoperative Infectious Complications After Gastric Cancer Surgery: An Analysis of Patients Enrolled in a Prospective Randomized Trial. Annals of Surgical Oncology. 2017;24:2688-97.
43. Magalhaes R, Magalhaes J, Pinto B, Curdia Goncalves T, Rosa B, Marinho C, et al. Neutrophil-to-lymphocyte ratio: An accurate method for predicting infection in cirrhosis. United European Gastroenterology Journal. 2018;6:A347.
44. Maimaiti Z, Xu C, Fu J, Chai W, Zhou Y, Chen J. The Potential Value of Monocyte to Lymphocyte Ratio, Platelet to Mean Platelet Volume Ratio in the Diagnosis of Periprosthetic Joint Infections. Orthopaedic Surgery. 2021;14:306-14.
45. Manuli E, Intra J, Limonta G, Cappellini F, Brambilla P. Risk estimate and features of infectious events in subjects with neutropenia. Biochimica Clinica. 2018;42:S77.
46. Marik PE, Stephenson E. The ability of Procalcitonin, lactate, white blood cell count and neutrophil-lymphocyte count ratio to predict blood stream infection. Analysis of a large database. Journal of Critical Care. 2020;60:135-9.
47. Markar SR, Karthikesalingam A, Falzon A, Kan Y. The diagnostic value of neutrophil: lymphocyte ratio in adults with suspected acute appendicitis. Acta Chirurgica Belgica. 2010;110(5):543-7.
48. Marrie TJ, Wu L. Factors influencing in-hospital mortality in community-acquired pneumonia: A prospective study of patients not initially admitted to the ICU. Chest. 2005;127:1260-70.
49. Masbang AN, Rosario MCO. Predictive value of white blood cell count and neutrophil-to-lymphocyte count ratio in classifying the severity of community acquired pneumonia in immunocompetent patients. Phillippine Journal of Internal Medicine. 2019;57:66-72.
50. McCluney SJ, Giakoustidis A, Segler A, Bissel J, Valente R, Hutchins RR, et al. Neutrophil: Lymphocyte ratio as a method of predicting complications following hepatic resection for colorectal liver metastasis. Journal of Surgical Oncology. 2018;117:1058-65.
51. Mehta AA, Anil Kumar V, Nair SG, Joseph FK, Kumar G, Singh SK. Clinical profile of patients admitted with swine-origin influenza a (H1N1) virus infection: An experience from a tertiary care hospital. Journal of Clinical and Diagnostic Research. 2013;7:2227-30.
52. Meka M, Raveesha A, Kalyani R. Prognostic Importance of Red Cell Distribution Width, Mean Platelet Volume and Neutrophil Lymphocyte Ratio among Sepsis Patients at a Tertiary Setting in Kolar, South India. Journal of Clinical and Diagnostic Research. 2022;16:EC23-EC7.
53. Miao YL, Zhang MX, Nie YL, Zhao W, Huang B, Jiang ZM, et al. Changes in T lymphocyte subsets after severe traumatic brain injury. Neural Regeneration Research. 2007;2:126-8.
54. Mohri Y, Tanaka K, Toiyama Y, Ohi M, Yasuda H, Inoue Y, et al. Impact of preoperative neutrophil to lymphocyte ratio and postoperative infectious complications on survival after curative gastrectomy for gastric cancer: A single institutional cohort study. Medicine (United States). 2016;95.
55. Moreau N, Wittebole X, Fleury Y, Forget P, Laterre PF, Castanares-Zapatero D. Neutrophil-to-Lymphocyte Ratio Predicts Death in Acute-on-Chronic Liver Failure Patients Admitted to the Intensive Care Unit: A Retrospective Cohort Study. Shock. 2017;49:385-92.
56. Moretto E, Cury V, Antoniazzi L, Oliveira PH, Borelli W, Cunha S, et al. Developing the pneumonia-optimized ratio for community-acquired pneumonia: an easy, inexpensive and accurate prognostic biomarker. European Respiratory Journal Conference: International Congress of the European Respiratory Society, ERS. 2021;58.
57. Nam KW, Kim TJ, Lee JS, Kwon HM, Lee YS, Ko SB, et al. High Neutrophil-to-Lymphocyte Ratio Predicts Stroke-Associated Pneumonia. Stroke. 2018;49(8):1886-92.
58. Nestor D, Andersson H, Kihlberg P, Olson S, Ziegler I, Rasmussen G, et al. Early prediction of blood stream infection in a prospectively collected cohort. BMC Infectious Diseases. 2021;21(1):316.
59. Nguyen J, Ferguson S, Bernardini M, May T, Laframboise S, Hogen L, et al. Preoperative neutrophil-to-lymphocyte ratio: A predictor of 30-day postoperative complications after primary surgery for ovarian cancer. International Journal of Gynecological Cancer. 2018;28:60.
60. Ni J, Wang H, Li Y, Shu Y, Liu Y. Neutrophil to lymphocyte ratio (NLR) as a prognostic marker for in-hospital mortality of patients with sepsis: A secondary analysis based on a single-center, retrospective, cohort study. Medicine. 2019;98:e18029.
61. Nishibe T, Kano M, Maekawa K, Matsumoto R, Fujiyoshi T, Iwahashi T, et al. Association of neutrophils, lymphocytes, and neutrophil-lymphocyte ratio to overall mortality after endovascular abdominal aortic aneurysm repair. International Angiology. 2022;41:136-42.
62. Okamura K, Nagata N, Wakamatsu K, Yonemoto K, Ikegame S, Kajiki A, et al. Hypoalbuminemia and lymphocytopenia are predictive risk factors for in-hospital mortality in patients with tuberculosis. Internal Medicine. 2013;52:439-44.
63. Ozmen I, Karakurt Z, Salturk C, Kargin F, Takir HB, Aksoy E, et al. Can N-terminal pro B-type natriuretic peptide, neutrophil-to-lymphocyte ratio, C-reactive protein help to predict short and long term mortality? Bratislavske Lekarske Listy. 2016;117(10):587-94.
64. Patel B, Oye M, Norez D, Isache C. Peripheral blood lymphocyte-to-monocyte ratio as a screening marker for influenza infection. Journal of Investigative Medicine. 2021;69:47-51.
65. Piotrowski D, Saczewska-Piotrowska A, Jaroszewicz J, Boron-Kaczmarska A. Lymphocyte-To-Monocyte Ratio as the Best Simple Predictor of Bacterial Infection in Patients with Liver Cirrhosis. International Journal of Environmental Research & Public Health [Electronic Resource]. 2020;17(5):06.
66. Polilli E, Esposito JE, Frattari A, Trave F, Sozio F, Ferr, et al. Circulating lymphocyte subsets as promising biomarkers to identify septic patients at higher risk of unfavorable outcome. BMC Infectious Diseases. 2021;21.
67. Ravindhran B, Hema AM, Vijaykumar M. Surgical site infection and antimicrobial resistance following resection and reconstructive surgery for oral cancers: Are we ready for the super bugs? British Journal of Surgery. 2021;108:i27.
68. Ren Y, Zhang L, Xu F, Han D, Zheng S, Zhang F, et al. Risk factor analysis and nomogram for predicting in-hospital mortality in ICU patients with sepsis and lung infection. BMC Pulmonary Medicine. 2021;22.
69. Rice J, Dodge JL, Bajaj JS, Reddy KR, Burton JR, Gralla J, et al. Admission neutrophil to lymphocyte ratio (NLR) is an independent predictor of mortality in hospitalized cirrhotics. Hepatology. 2017;66:294A.
70. Rice J, Dodge JL, Bambha KM, Bajaj JS, Reddy KR, Gralla J, et al. Neutrophil-to-Lymphocyte Ratio Associates Independently With Mortality in Hospitalized Patients With Cirrhosis. Clinical Gastroenterology and Hepatology. 2018;16:1786-91.e1.
71. Riche F, Gayat E, Barthelemy R, Le Dorze M, Mateo J, Payen D. Reversal of neutrophil-to-lymphocyte count ratio in early versus late death from septic shock. Critical Care. 2015;19:439.
72. Rovera F, Imperatori A, Militello P, Morri A, Antonini C, Dionigi G, et al. Infections in 346 consecutive video-assisted thoracoscopic procedures. Surgical Infections. 2003;4:45-51.
73. Salciccioli JD, Marshall DC, Pimentel MAF, Santos MD, Pollard T, Celi LA, et al. The association between the neutrophil-to-lymphocyte ratio and mortality in critical illness: An observational cohort study. Critical Care. 2015;19.
74. Salturk C, Karakurt Z, Adiguzel N, Kargin F, Sari R, Celik ME, et al. Does eosinophilic COPD exacerbation have a better patient outcome than non-eosinophilic in the intensive care unit? International Journal of COPD. 2015;10:1837-46.
75. Sbeit W, Kadah A, Shahin A, Abed N, Haddad H, Jabbour A, et al. Predictors of in-hospital mortality among patients with clostridium difficile infection: A multicenter study. Minerva Medica. 2021;112:124-9.
76. Schietroma M, Piccione F, Carlei F, Clementi M, Bianchi Z, De Vita F, et al. Peritonitis from perforated appendicitis: Stress response after laparoscopic or open treatment. American Surgeon. 2012;78:582-90.
77. Schroeder S, Lindemann C, Decker D, Klaschik S, Hering R, Putensen C, et al. Increased susceptibility to apoptosis in circulating lymphocytes of critically ill patients. Langenbeck's Archives of Surgery. 2001;386:42-6.
78. Sen P, Demirdal T, Nemli SA, Vardar I, Kizilkaya M, Sencan A, et al. Infection markers as predictors of bacteremia in an intensive care unit: A prospective study. Pakistan Journal of Medical Sciences. 2018;34:1517-24.
79. Serban D, Papanas N, Dascalu AM, Kempler P, Raz I, Rizvi AA, et al. Significance of Neutrophil to Lymphocyte Ratio (NLR) and Platelet Lymphocyte Ratio (PLR) in Diabetic Foot Ulcer and Potential New Therapeutic Targets. International Journal of Lower Extremity Wounds. 2021.
80. Shadrivova OV, Shevyakov MA, Desyatik EA, Ignatyeva SM, Zjuzgin IS, Chudinovskih JA, et al. Abdominal organs involvement in patients with invasive aspergillosis: Results of the multicenter study in Saint Petersburg, Russia. Medical Mycology. 2018;56:S74.
81. Shankar-Hari M, Datta D, Wilson J, Assi V, Stephen J, Weir CJ, et al. Early PREdiction of sepsis using leukocyte surface biomarkers: the ExPRES-sepsis cohort study. Intensive Care Medicine. 2018;44:1836-48.
82. Sharma SK, Mohan A, Banga A, Saha PK, Guntupalli KK. Predictors of development and outcome in patients with acute respiratory distress syndrome due to tuberculosis. International Journal of Tuberculosis and Lung Disease. 2006;10:429-35.
83. Shen Y, Huang X, Zhang W. Platelet-to-lymphocyte ratio as a prognostic predictor of mortality for sepsis: Interaction effect with disease severity - A retrospective study. BMJ Open. 2019;9.
84. Shi K, Huang Y, Zhang Q, Li Y, Wang X. Neutrophil-lymphocyte ratio and the risk of 30-day mortality in patients with overt hepatic encephalopathy. European Journal of Gastroenterology and Hepatology. 2022;34:529-36.
85. Shlomai A, Nutman A, Kotlovsky T, Schechner V, Carmeli Y, Guzner-Gur H. Predictors of pandemic (H1N1) 2009 virus positivity and adverse outcomes among hospitalized patients with a compatible syndrome. Israel Medical Association Journal. 2010;12:622-7.
86. Soetjipto AS, Widysanto A, Stella Soetjipto A, Sungono V. Role of admission serum procalcitonin compared to neutrophil-lymphocyte count ratio for assessing severity and mortality in hospitalized community acquired pneumonia. Respirology. 2016;21:87.
87. Stortz JA, Murphy TJ, Raymond SL, Mira JC, Ungaro R, Dirain ML, et al. Evidence for persistent immune suppression in patients who develop chronic critical illness after sepsis. Shock. 2017;49:249-58.
88. Sun H, Que J, Peng Y, Ye H, Xiang H, Han Y, et al. The neutrophil-lymphocyte ratio: A promising predictor of mortality in coronary care unit patients - A cohort study. International Immunopharmacology. 2019;74.
89. Sun J, Guo H, Yu X, Zhu H, Zhang X, Yang J, et al. A neutrophil-to-lymphocyte ratio-based prognostic model to predict mortality in patients with HBV-related acute-on-chronic liver failure. BMC Gastroenterology. 2021;21.
90. Tapadia AK, Sameerkumar GS, Jain M, Balkrishnan M, Varghese J, Venkatatraman J. Utility of C-reactive protein (CRP) & neutrophil to lymphocyte ratio (NLR) as simple surrogate markers of infection & predicting outcome in hospitalized patients with liver cirrhosis. Journal of Gastroenterology and Hepatology. 2019;34:702.
91. Terradas R, Grau S, Blanch J, Riu M, Saballs P, Castells X, et al. Eosinophil count and neutrophil-lymphocyte count ratio as prognostic markers in patients with bacteremia: A retrospective cohort study. PLoS ONE. 2012;7.
92. Tian T, Wei B, Wang J. Study of C-reactive protein, procalcitonin, and immunocyte ratios in 194 patients with sepsis. BMC Emergency Medicine. 2021;21.
93. Tschaikowsky K, Hedwig-Geissing M, Schiele A, Bremer F, Schywalsky M, Schuttler J. Coincidence of pro- and anti-inflammatory responses in the early phase of severe sepsis: Longitudinal study of mononuclear histocompatibility leukocyte antigen-DR expression, procalcitonin, C-reactive protein, and changes in T-cell subsets in septic and postoperative patients. Critical Care Medicine. 2002;30:1015-23.
94. Visveswari G, Min B, Lateef F. Diagnostic value of biomarkers for sepsis in adult patients in the emergency department: Don't forget the neutrophil-lymphocyte count ratio. Journal of Acute Disease. 2019;8:45-52.
95. Wang Y, Huang X, Sun T, Fan G, Zhan Q, Weng L. Non-HIV-infected patients with Pneumocystis pneumonia in the intensive care unit: A bicentric, retrospective study focused on predictive factors of in-hospital mortality. Clinical Respiratory Journal. 2022;16:152-61.
96. Weijian E, Wang Z, Pang M, Lu Y, Fan H. The correlation between platelet-to-lymphocyte ratio and neutrophil-to-lymphocyte ratio with hepatic echinococcosis. Journal of Inflammation Research. 2021;14:2403-9.
97. Westerdijk K, Simons KS, Zegers M, Wever PC, Pickkers P, de Jager CPC. The value of the neutrophil-lymphocyte count ratio in the diagnosis of sepsis in patients admitted to the Intensive Care Unit: A retrospective cohort study. PLoS ONE [Electronic Resource]. 2019;14(2):e0212861.
98. Wyllie DH, Bowler IC, Peto TE. Relation between lymphopenia and bacteraemia in UK adults with medical emergencies. Journal of Clinical Pathology. 2004;57(9):950-5.
99. Wyllie DH, Bowler ICJW, Peto TEA. Bacteraemia prediction in emergency medical admissions: Role of C reactive protein. Journal of Clinical Pathology. 2005;58:352-6.
100. Xu H, Xie J, Zhang S, Wang D, Huang Z, Zhou Z. Potential Blood Biomarkers for Diagnosing Periprosthetic Joint Infection: A Single-Center, Retrospective Study. Antibiotics. 2022;11.
101. Zahorec R. Ratio of neutrophil to lymphocyte counts--rapid and simple parameter of systemic inflammation and stress in critically ill. Bratislavske lekarske listy. 2001;102:5-14.
102. Zencir C, Akpek M, Senol S, Selvi M, Onay S, Cetin M, et al. Association between hematologic parameters and in-hospital mortality in patients with infective endocarditis. Kaohsiung Journal of Medical Sciences. 2015;31:632-8.
103. Zhang HF, Ge YL, Wang HY, Zhang Q, Li WQ, Chen Y, et al. Neutrophil-to-Lymphocyte Ratio Improves the Accuracy and Sensitivity of Pneumonia Severity Index in Predicting 30-Day Mortality of CAP Patients. Clinical Laboratory. 2019;65(10):01.

**Outcome of interest not reported (n = 22)**

1. Chen L, Han X, Li Y, Zhang C, Xing X. Invasive pulmonary aspergillosis in immunocompetent patients hospitalised with influenza A-related pneumonia: A multicenter retrospective study. BMC Pulmonary Medicine. 2020;20.
2. Curbelo J, Bueno SL, Galvan-Roman JM, Ortega-Gomez M, Rajas O, Fern, et al. Inflammation biomarkers in blood as mortality predictors in community-acquired pneumonia admitted patients: Importance of comparison with neutrophil count percentage or neutrophil-lymphocyte ratio. PLoS ONE. 2017;12.
3. de Jager CPC, Wever PC, Gemen EFA, Kusters R, van Gageldonk-Lafeber AB, van der Poll T, et al. The Neutrophil-Lymphocyte Count Ratio in Patients with Community-Acquired Pneumonia. PLoS ONE. 2012;7.
4. Deng QW, Gong PY, Chen XL, Liu YK, Jiang T, Zhou F, et al. Admission blood cell counts are predictive of stroke-associated infection in acute ischemic stroke patients treated with endovascular therapy. Neurological Sciences. 2020;42:2397-409.
5. Elsorady KE. Predictors of Multi-Drug Resistant Gram-Negative Bacterial Infection in Critically Ill Older Adults. Aging Medicine and Healthcare. 2022;13:32-9.
6. Fu B, Wu Z, Huang L, Chai Z, Zheng P, Sun Q, et al. A comparison of demographic, epidemiological and clinical characteristics of hospital influenza-related viral pneumonia patients. BMC Infectious Diseases. 2021;21.
7. Gong Y, Li C, Wang C, Li J, Ding M, Chen D, et al. Epidemiology and mortality-associated factors of invasive fungal disease in elderly patients: A 20-year retrospective study from Southern China. Infection and Drug Resistance. 2020;13:711-23.
8. Greene KA, Wilde AH, Stulberg BN. Preoperative nutritional status of total joint patients: Relationship to postoperative wound complications. Journal of Arthroplasty. 1991;6:321-5.
9. Korkmaz P, Erarslan S, Toka O. Evaluation of the association between the neutrophil to lymphocyte ratio and mortality in the patients followed up with the diagnosis of sepsis. Journal of Clinical and Analytical Medicine. 2017;8:211-5.
10. Lee DU, Alvencar S, Beano A, Seo DJ, Fan GH, Wang E, et al. Lymphocytic count as a biomarker that predicts portal inflammation in liver transplant patients. American Journal of Gastroenterology. 2020;115:S527.
11. Li D, Zhang J, Bai G, Chen J, Cheng W, Cui N. Lymphocyte and NK Cell Counts Can Predict Sepsis-Associated Delirium in Elderly Patients. Frontiers in Aging Neuroscience. 2021;12.
12. Li L, Wang C, Sun L, Zhang X, Yang G. Clinical characteristics and prognostic risk factors of mortality in patients with interstitial lung diseases and viral infection: A retrospective cohort study. Journal of Medical Microbiology. 2021;70.
13. Lv J, Zong H, Ma G, Wei X, Zhao Y, Wang Q. Predictive significance of peripheral blood smears in patients with fever of unknown origin: A retrospective study of 2871 cases. Clinical Laboratory. 2015;61:1643-52.
14. Maruyama Y, Inoue K, Mori K, Shimamoto R, Onizuka T, Okazaki M, et al. Peripheral blood parameters as novel predictors of postoperative wound healing failure in head and neck reconstruction. Journal of Wound Care. 2017;26:182.
15. Mene-Afejuku TO, Kim KS, Akinlonu A, Ngo EM, Salazar P, Perez JA, et al. The Prognostic Value of Complete Blood Count among Patients with Acute Decompensated Heart Failure. Journal of Cardiac Failure. 2019;25:S147.
16. Merdler I, Frydman S, Sirota S, Halkin A, Steinvil A, Toledano E, et al. Neutrophil-to-Lymphocyte Ratio as a Prognostic Marker in Transcatheter Aortic Valve Implantation (TAVI) Patients. Israel Medical Association Journal. 2022;24:229-34.
17. Moz M, Araujo C, Laszcznska O, Margato R, Maciel MJ, Moreira I, et al. Differences in inflammatory markers by type of acute coronary syndrome and its relation with severity and outcome. Revista Portuguesa de Cardiologia. 2016;35:103.
18. Naess A, Nilssen SS, Mo R, Eide GE, Sjursen H. Role of neutrophil to lymphocyte and monocyte to lymphocyte ratios in the diagnosis of bacterial infection in patients with fever. Infection. 2016;45:299-307.
19. Pacelli F, Doglietto GB, Alfieri S, Piccioni E, Sgadari A, Gui D, et al. Prognosis in intra-abdominal infections: Multivariate analysis on 604 patients. Archives of Surgery. 1996;131:641-5.
20. Pfitzenmeyer P, Decrey H, Auckenthaler R, Michel JP. Predicting bacteremia in older patients. Journal of the American Geriatrics Society. 1995;43:230-5.
21. Sadamatsu H, Takahashi K, Tashiro H, Kusaba K, Haraguchi T, Kurihara Y, et al. A low body mass index is associated with unsuccessful treatment in patients with mycobacterium avium complex pulmonary disease. Journal of Clinical Medicine. 2021;10.
22. Singh H, Cotarelo A, Samarneh MM, Rampersaud R. 289 Neutrophil to Lymphocyte Ratio and Platelet to Lymphocyte Ratio as Predictive Markers for Pulmonary Embolism. Annals of Emergency Medicine. 2020;76:S111-S2.

**Incorrect study population (n = 27)**

1. Chen L, Han X, Li YL, Zhang C, Xing X. The severity and risk factors for mortality in immunocompromised adult patients hospitalized with influenza-related pneumonia. Annals of Clinical Microbiology and Antimicrobials. 2021;20.
2. Dujardin A, Lorent M, Kerleau C, Brouard S, Giral M. Post-transplant lymphopenia is associated with higher risk of graft failure, death and viral infections. American Journal of Transplantation. 2019;19:756-7.
3. Goupil R, Brachemi S, Nadeau ACF, Deziel C, Troyanov Y, Lavergne V, et al. Lymphopenia and treatment-related infectious complications in ANCA-associated vasculitis. Clinical Journal of the American Society of Nephrology. 2013;8:416-23.
4. He T, Wu Y, Li X, Yang M, Lin Q. Risk factors for infection-related hospitalization in end-stage renal disease patients during peri-dialysis period. Therapeutic Apheresis and Dialysis. 2021;26:717-25.
5. Joshi AY, Boyce TG. Lymphopenia is a risk factor for pneumonia and hospitalization in down syndrome. Annals of Allergy, Asthma and Immunology. 2010;105:A98.
6. Karimi F, Ashrafi F, Moghaddas A, Derakhsheh A. Management of febrile neutropenia: A description of clinical and microbiological findings by focusing on risk factors and pitfalls. Journal of Research in Pharmacy Practice. 2018;7:147-56.
7. Luo D, Li H, Yu H, Zhang M, Hu J, Jin C, et al. Predictive value of preoperative and postoperative peripheral lymphocyte difference in hepatitis B virus-related hepatocellular cancer patients: Based on the analysis of dynamic nomogram. Journal of Surgical Oncology. 2020;122:1553-68.
8. Morey VM, Song YD, Whang JS, Kang YG, Kim TK. Can Serum Albumin Level and Total Lymphocyte Count be Surrogates for Malnutrition to Predict Wound Complications After Total Knee Arthroplasty? Journal of Arthroplasty. 2015;31:1317-21.
9. Muratoglu M, Kayipmaz AE, Kavalci C, Kirnap M, Moray G, Haberal M. Platelet-to-Lymphocyte Ratio as a Potential Indicator of Infection-Associated Emergency Visits of Renal Transplant Recipients. Experimental and clinical transplantation : official journal of the Middle East Society for Organ Transplantation. 2019;9.
10. Muto R, Kato S, Lindholm B, Qureshi AR, Ishimoto T, Kosugi T, et al. Increased Monocyte/Lymphocyte Ratio as Risk Marker for Cardiovascular Events and Infectious Disease Hospitalization in Dialysis Patients. Blood Purification. 2021.
11. Nierenberg NE, Poutsiaka DD, Chow JK, Cooper J, Price LL, Freeman RB, et al. Pretransplant lymphopenia is a novel prognostic factor in cytomegalovirus and noncytomegalovirus invasive infections after liver transplantation. Liver Transplantation. 2014;20:1497-507.
12. Nino MC, Cohen D, Aguilar P, Rodriguez EE, Madrid GA. Intravenous lidocaine during craniotomy does not blunt inflammatory response to surgery. Critical Care Conference: 41st International Symposium on Intensive Care and Emergency Medicine Brussels Belgium. 2022;26.
13. Octeau D, Barnes HJ, Faries CM, Nakazawa KR, Ting W, Marin ML, et al. Association of Preoperative Neutrophil-to-Lymphocyte Ratio With Rates of Adverse Events After Thoracic Endovascular Aneurysm Repair. Journal of Vascular Surgery. 2020;72:e186.
14. Pang J, Thein TL, Leo YS, Lye DC. Early clinical and laboratory risk factors of intensive care unit requirement during 2004-2008 dengue epidemics in Singapore: A matched case-control study. BMC Infectious Diseases. 2014;14.
15. Quintanilla-Gonzalez L, Torres-Villalobos G, Hinojosa-Azaola A. Risk factors for development of early infectious and noninfectious complications in systemic lupus erythematosus patients undergoing major surgery. Lupus. 2018;27:1960-72.
16. Rajas O, Ortega-Gomez M, Galvan Roman JM, Curbelo J, Fern, ez Jimenez G, et al. The incidence of cardiovascular events after hospitalization due to CAP and their association with different inflammatory markers. BMC Pulmonary Medicine. 2014;14:197.
17. Ray-Coquard I, Borg C, Bachelot T, Sebban C, Philip I, Clapisson G, et al. Baseline and early lymphopenia predict for the risk of febrile neutropenia after chemotherapy. British Journal of Cancer. 2003;88:181-6.
18. Sarin S, Pamecha V, Sinha PK, Patil N, Mahapatra N. Neutrophil Lymphocyte Ratio can Preempt Development of Sepsis After Adult Living Donor Liver Transplantation. Journal of Clinical and Experimental Hepatology. 2021;12:1142-9.
19. Schietroma M, Colozzi S, Pessia B, Carlei F, Amicucci G. The Effects of High-Concentration Oxygen on Inflammatory Markers in Laparoscopic Cholecystectomy: A Randomized Controlled Trial. Surgical Laparoscopy, Endoscopy and Percutaneous Techniques. 2017;27:83-9.
20. Schoeberl A, Kaider A, Goekler J, Zuckermann AO. Absolute Lymphocyte Count as a Prognostic Factor for Cytomegalovirus Infection After Heart Transplantation. Journal of Heart and Lung Transplantation. 2022;41:S280.
21. Shadrivova O, Desyatik E, Volkova A, Popova M, Uspenskaya O, Shneyder T, et al. Invasive aspergillosis in elderly oncohematological patients. HemaSphere. 2019;3:917-8.
22. Silagy AW, Tin AL, Rappold P, Vertosick EA, Mano R, Attalla K, et al. Systemic Immunological Determinants of Oncological Outcomes After Surgery for Localized Renal Cell Carcinoma. Clinical Genitourinary Cancer. 2022.
23. Slim M, Sanabria J, Robles-Diaz M, Medina-Caliz I, Gonzalez-Jimenez A, Ortega A, et al. Clinical characteristics and outcomes of elderly included in the Spanish Drug-Induced Liver Injury (DILI) Registry. Journal of Hepatology. 2017;66:S399.
24. Tagawa M, Nishimoto M, Kokubu M, Matsui M, Eriguchi M, Samejima KI, et al. Acute kidney injury as an independent predictor of infection and malignancy: the NARA-AKI cohort study. Journal of Nephrology. 2019;32:967-75.
25. Tani M, Iida H, Maehira H, Mori H, Miyake T, Kaida S. A High C-Reactive Protein Level on Postoperative Day 7 is Associated with Poor Survival of Patients with Pancreatic Ductal Adenocarcinoma after Resection. American Surgeon. 2021.
26. Tartar AS, Balin SO. Geriatric urinary tract infections: The value of laboratory parameters in estimating the need for bacteremia and intensive care unit. Pakistan Journal of Medical Sciences. 2019;35:215-9.
27. Teerasarntipan T, Chaiteerakij R, Komolmit P, Treeprasertsuk S. Predictors of acute liver failure and death among patients with dengue-induced severe hepatitis. Journal of Hepatology. 2020;73:S228.

**Conference abstract or Editorial (n = 53)**

1. Arikan H, Ozdemir C, Karakurt S. The association between neutrophil-to-lymphocyte ratio and mortality in critically ill patients: A retrospective cohort study. Turkish Thoracic Journal. 2019;20:S12.
2. Attanasio L, Grimaldi D, Akhtar Ramiz R, Schuind S, Creteur J, Spadaro S, et al. Early lymphopenia and infections in non-traumatic subarachnoid hemorrhage. Intensive Care Medicine Experimental Conference: 32nd European Society of Intensive Care Medicine Annual Congress, ESICM. 2019;7.
3. Aurelien F, Mathieu L, Murielle G, Fabrice U, Arnaud G, Adel M, et al. ECLS induces changes in immune cells that may promote nosocomial infection. Annals of Intensive Care Conference: French Intensive Care Society, International Congress Reanimation. 2018;8(1).
4. Barbosa MS, Caldas J, Melo N, Ferreira A, Garcia D, Lourenco P. Predictors of in-hospital mortality in influenza infection. European Respiratory Journal Conference: 29th International Congress of the European Respiratory Society, ERS Madrid Spain. 2019;54.
5. Bellelli V, D'Ettorre G, Celani L, Borrazzo C, Ceccarelli G, Venditti M. Clinical significance of lymphocytopenia in patients hospitalized with pneumonia caused by influenza virus. Critical Care. 2019;23.
6. Brakenridge SC, Lysak N, Ghita G, Wang Z, Brumback B, Ozrazgat-Baslanti T, et al. Comparison of sepsis-2 and sepsis-3 clinical criteria in critically ill patients: Is there any impact on discrimination of immunophenotype and clinical outcomes? Shock. 2018;49:138-9.
7. Campos CC, Dominedo C, Gabarrus A, Garcia-Vidal C, Moreno E, Vargas CR, et al. Methicillin-susceptible Staphylococcus aureus in Community-Acquired Pneumonia: Risk Factors and Outcomes. European Respiratory Journal Conference: European Respiratory Society International Congress, ERS. 2020;56.
8. Chien YC, Chung KP, Cheng JS, Chang HT, Yu CJ. Lymphopenia is associated with worse outcome in patients with severe sepsis. American Journal of Respiratory and Critical Care Medicine Conference: American Thoracic Society International Conference, ATS. 2012;185.
9. Cohen JT, Charpentier KP, Miner TJ, Cioffi WG, Beard RE. Persistent lymphopenia following pancreaticoduodenectomy predicts clinically relevant pancreatic fistula formation. Hpb. 2020;22:S118.
10. Contreras Contreras AR, Najera E, Pedraza A, Franco J, Aguirre J, Camarena G. Clinical impact of body mass index and lymphocytic count on clinical outcomes in septic patients in a critical care unit: Chronic inflammation as a protective factor? Intensive Care Medicine Experimental Conference: 31st European Society of Intensive Care Medicine Annual Congress, ESICM. 2018;6.
11. Damiani E, Domizi R, Scorcella C, Tondi S, Pierantozzi S, Ciucani S, et al. Neutrophil-lymphocyte ratio and mortality during critical illness. Critical Care Conference: 37th International Symposium on Intensive Care and Emergency Medicine Brussels Belgium. 2017;21(1).
12. De Pastena M, Fumagalli L, Paiella S, Malleo G, Marchegiani G, Salvia R, et al. Low postoperative lymphocyte count as negative predictor of major complications in pancreatic resections. Pancreatology. 2016;16:S80.
13. Deek AJ, Scott CA, Fahmy MD, Hiedel REE, Clegg D, Hudson JW, et al. Pre-operative Lab Values and Wound Healing following Facial Trauma Surgery: Which Values Predict Postoperative Complications? Journal of Oral and Maxillofacial Surgery. 2021;79:e103-e4.
14. Dosanjh DPS, Grudzinska FS, Aldridge K, Nightingale P, Hughes S, Thickett D. Factors independently associated with poor outcomes in community acquired pneumonia-a multivariable analysis. Thorax. 2018;73:A107-A8.
15. Dupont G, Flory L, Tourel C, Morel J, Mor, D, et al. Assessment of postoperative lymphopenia as risk factor for postoperative infections: EVALYMPH study. Intensive Care Medicine Experimental Conference: 31st European Society of Intensive Care Medicine Annual Congress, ESICM. 2018;6.
16. Efron P, Stortz J, Horiguchi H, Hollen M, Weiss B, Mohr A, et al. The immune status and outcomes of septic patients who develop secondary infections. Critical Care Medicine. 2018;46:715.
17. Ferrari M, Keegan G, Williams K, Welters ID. Prognostic role of neutrophil lymphocyte ratio (NLR) in critical illness. Critical Care Conference: 38th International Symposium on Intensive Care and Emergency Medicine, ISICEM. 2018;22.
18. Greene KA, Wilde AH, Stulberg BN. Preoperative nutritional status of total joint patients: Relationship to postoperative wound complications. Journal of Arthroplasty. 1991;6:321-5.
19. Guven Azap ME, Yalaz Tekan U, Pirdal BZ, Necioglu Orken D. Risk factors, management and inpatient outcomes of pneumonia after acute stroke. International Journal of Stroke. 2020;15:104.
20. Hasegawa E, Kobayashi D, Ito S, Narita I, Nakazono K. The risk factors of serious infection in patients with rheumatoid arthritis. Annals of the Rheumatic Diseases. 2018;77:1061.
21. Hung IFN, Li J, To KK, Tam A, Chan JF. High mortality associated with parainfluenza virus infection in hospitalized adults. Open Forum Infectious Diseases. 2014;4:S568.
22. Itoh A, Kudo A, Kawakami K, Nishimura K, Takano Y, Inanami H. The incidence and risk factors of surgical site infection in micro endoscope-assisted posterior lumbar interbody fusion. Journal of Orthopaedic Research Conference. 2017;35.
23. Iturriaga LAR, Fern, ez LS, Quero BG, Bonilla AG, Fern, et al. Lymphopenic community acquired pneumococcal pneumonia: Clinical characteristics and outcome. European Respiratory Journal Conference: International Congress of the European Respiratory Society, ERS. 2021;58.
24. Iwata E, Yamamoto Y, Shigematsu H, Nakajima H, Tanaka M, Okuda A, et al. Lymphocyte count at 4 days postoperatively and CRP level at 7 days postoperatively: Reliable and useful markers for surgical site infection following instrumented spinal fusion. European Spine Journal. 2018;27:S640.
25. Keegan GL, Welters I. Significance of Neutrophil Lymphocyte Ratio in outcome of sepsis. Intensive Care Medicine Experimental Conference: 30th Annual Congress of the European Society of Intensive Care Medicine, ESICM. 2017;5(2).
26. Kim TJ, Lee CH, Mo H, Jeong HY, Ko SB, Yoon BW. Neutrophil-to-lymphocyte ratio is a useful predictor of aspiration pneumonia in patients with acute ischemic stroke. Stroke Conference: American Heart Association/American Stroke Association. 2018;49.
27. Krishnan J, Nachar V, Marini BL, Perissinotti AJ, Phillips TJ, Kaminski MS, et al. Risk of Opportunistic Infections in Patients with Bendamustine-Associated Lymphopenia and the Utility of Antimicrobial Prophylaxis. Blood. 2020;136:50.
28. Lazovic B, Milic R, Zugic V. The neutrophil-lymphocyte count ratio in patients with hospital-acquired pneumonia. European Respiratory Journal Conference: 29th International Congress of the European Respiratory Society, ERS Madrid Spain. 2019;54.
29. Lee DU, Fan GH, Karagozian R. The effect of cirrhosis on the outcomes of hospitalized patients with influenza infection: A nationwide analysis. American Journal of Gastroenterology. 2021;115:S524.
30. Lu Z, Li J, Cao H, Xia Y, Chen Y, Guo G, et al. Average lymphocyte count, an useful marker for the prognosis in systemic lupus erythematosus patients with pulmonary infection. International Journal of Rheumatic Diseases. 2019;21:142-3.
31. Lugg S, Agostini P, Kerr A, Kalkat M, Rajesh P, Steyn R, et al. The role of lymphopenia in the development and severity of postoperative pulmonary complications after lung surgery. European Respiratory Journal Conference: European Respiratory Society International Congress, ERS. 2017;50.
32. Mahmoud HO, Fahmy NG, Mohamed Abdelmotaleb Ammar MA, Mohamed Elsaid EMM. Neutrophil-to-lymphocyte count ratio in predicting prognosis of septic shock patients. QJM Conference: 41st Annual International Ain Shams Medical Congress Online. 2021;114.
33. Maniar N, Cavallazzi R. Predictors of bacteremia from the complete blood count in patients in the intensive care unit. American Journal of Respiratory and Critical Care Medicine Conference: American Thoracic Society International Conference, ATS. 2017;195.
34. Mansour W, Karam B, Zaidan J, Kfoury B, Abbasi S, El Bitar S, et al. Neutrophil-to-lymphocyte ratio as predictor for hospital mortality in patients with acute pulmonary embolism. American Journal of Respiratory and Critical Care Medicine Conference: American Thoracic Society International Conference, ATS. 2018;197.
35. Martinelli A, Pagani G, Conte G, Bertu L, Turato S, Grazioli S, et al. Independent role of white blood cells in predicting the short term outcome in patients with acute pulmonary embolism. Italian Journal of Medicine. 2016;10:73-4.
36. Mendez R, Amara-Elori I, Feced L, Ramirez P, Bermejo-Martin JF, Sempere A, et al. Lymphocyte subpopulations in community-acquired pneumonia: Role of cell-mediated immunity. American Journal of Respiratory and Critical Care Medicine Conference: American Thoracic Society International Conference, ATS. 2018;197.
37. Meyer N, Wu L, Agyekum RS, Dunn TG, Ittner CCAG, Weisman A, et al. Absolute lymphopenia associates with plasma markers of hyperinflammation. American Journal of Respiratory and Critical Care Medicine Conference: American Thoracic Society International Conference, ATS. 2020;201(1).
38. Moz M, Laszczynska O, Araujo C, Margato R, Maciel MJ, Moreira JI, et al. Inflammation, atrial fibrillation and ventricular arrhythmias in acute coronary syndrome patients. European Heart Journal. 2016;37:187.
39. Neeser O, Vukajlovic T, Felder L, Haubitz S, Hammerer-Lercher A, Ottiger C, et al. A high C-reactive protein/procalcitonin ratio predicts Mycoplasma pneumoniae infection. Critical Care Conference: 39th International Symposium on Intensive Care and Emergency Medicine Brussels Belgium. 2019;23.
40. Nguyen J, Paleri S, Baradi A, Ellis Z, Huang K, Navani R, et al. Evaluation of Haematological and Biochemical Markers as Simple Predictors of In-hospital Mortality in Infective Endocarditis. Heart Lung and Circulation. 2019;28:S297.
41. Ni YX, Gu C, Zhu L. Lymphocytopenia and clinical performance of acute physiology and chronic health evaluation IV in severe community-acquired pneumonia. American Journal of Respiratory and Critical Care Medicine Conference. 2019;199(9).
42. Nseir W, Khamisy-Farah R, Amara A, Farah R. The prognostic value of inflammatory markers in clostridium difficile-associated diarrhea. Israel Medical Association Journal. 2019;21:658-61.
43. Parke AP, Yu DY, Unge CU, Sunden-Cullberg JC. Plasma-calprotectin compared with routine biomarkers for prediction of early severe event in sepsis. Critical Care Conference: 40th International Symposium on Intensive Care and Emergency Medicine Brussels Belgium. 2020;24.
44. Pena C, Costi AC, Pera M, Garcia N, Garcia L, Savy F, et al. Severe infections in systemic necrotizing vasculitis: Incidence and risk factors. Journal of Clinical Rheumatology. 2017;25:S87.
45. Pires MI, Goncalves ML, Antunes H, Goncalves Pereira JG, Santos JM, Costa Cabral J, et al. The use of neutrophil-to-lymphocyte ratio and platelet-to-lymphocyte ratio in the prognosis of acute coronary syndromes. European Heart Journal: Acute Cardiovascular Care. 2019;8:364.
46. Pollock A, Galbraith N, Bleakley A, Setford I, Halcrow E, Moug S. Lymphopaenia and laparotomy-a predictor of mortality. British Journal of Surgery. 2021;108:vii209.
47. Purcarea A, Sovaila S, Bros A, Sauleau E, Andres E, Bourgarit A. The neutrophil index-Lymphocyte and its usefulness in the early diagnosis of infection in elderly patients. Cohort study. European Journal of Internal Medicine. 2013;1:e197-e8.
48. Sedghiani I, Benabderrahim A, Hamdi D, Jendoubi A, Cherif MA, Hechmi YZE, et al. Sepsis and septic shock: Prognostic value of lymphocytopenia. Annals of Intensive Care. 2017;7:152-3.
49. Sen P, Demirdal T, Nemli A, Vardar I, Kizilkaya M, Sencan A. The significance of neutrophil to lymphocyte ratio and the other infection markers on predicting bacteremia and prognosis in patients diagnosed with systemic inflammatory response syndrome (SIRS) and sepsis in intensive care unit. Open Forum Infectious Diseases Conference: ID Week. 2016;3.
50. Sheth R, Bhatia M, Kak V. Clinical Predictors of Hospital-Acquired Bloodstream Infections. Open Forum Infectious Diseases. 2021;8:S423.
51. Slajus B, Brailovsky Y, Phan T, Darwish I, Fareed J, Darki A. Predictive value of cellular blood indices for all-cause mortality in acute pulmonary embolism. Blood Conference: 61st Annual Meeting of the American Society of Hematology, ASH. 2019;134.
52. Suarez-Cuenca J, Ruiz-Hern, ez A, Corona-Rojas L, Flores-Zaleta J, Vera-Gomez E, et al. Role of neutrophil-to-lymphocyte ratio in the prognosis of severe community-acquired pneumonia. Intensive Care Medicine Experimental Conference: 31st European Society of Intensive Care Medicine Annual Congress, ESICM. 2018;6.
53. Wu H. The Value of Neutrophil to Lymphocyte Count Ratio for Predicting the Clinical Outcomes of Patients with Carbapenem-resistant Klebsiella pneumonia Blood Stream Infection. Open Forum Infectious Diseases. 2021;8:S219.

**Duplicate study/population (n=12)**

1. Calcagno J, Kumar R, Maza A, Awan N, Ferimer S, Nierstedt R, et al. Lymphopenia after traumatic brain injury increases infections, hospital resource utilization, and worsens long-term outcomes. Journal of Neurotrauma. 2018;35:A135-A6.
2. Ceccato A, Panagiotarakou M, Martin-Fern, ez M, Mora RA, Ranzani O, et al. LYMPHOPENIA AS PREDICTOR of LONG TERM MORTALITY in PATIENTS with HOSPITAL ACQUIRED PNEUMONIA. European Respiratory Journal Conference: European Respiratory Society International Congress, ERS. 2018;52.
3. Drewry A, Skrupky L, Fuller B, Hotchkiss R. Hypothermia on the day of sepsis diagnosis predicts persistent lymphopenia. Critical Care Medicine. 2016;1:A18.
4. Drewry AM, Fuller BM, Skrupky LP, Hotchkiss RS. The Presence of Hypothermia Within 24 Hours of Sepsis Diagnosis Predicts Persistent Lymphopenia. Critical Care Medicine. 2015;43:1165-9.
5. Gong YY, Ding ML, Chen L, Li J, Lao MX. Clinical analysis of invasive fungal diseases in elderly patients from southern China. Journal of the American Geriatrics Society. 2020;67:S674-S7.
6. Inose H, Kobayashi Y, Yuasa M, Hirai T, Yoshii T, Okawa A. Procalcitonin and Neutrophil Lymphocyte Ratio After Spinal Instrumentation Surgery. Spine. 2019;44(23):E1356-E61.
7. Iwata E, Shigematsu H, Yamamoto Y, Tanaka M, Okuda A, Morimoto Y, et al. Lymphocyte Count at 4 Days Postoperatively: A Reliable Screening Marker for Surgical Site Infection After Posterior Lumbar Decompression Surgery. Spine. 2016;43(18):E1096-E101.
8. Iwata E, Shigematsu H, Yamamoto Y, Tanaka M, Okuda A, Morimoto Y, et al. Lymphocyte count at 4 days postoperatively. Spine. 2018;43:E1096-E101.
9. Levochkina M, Vaughan L, Awan N, Wagner A. Temporal dynamics of neutrophil-to-lymphocyte levels and infections after traumatic brain injury. Journal of Neurotrauma. 2021;38:A15.
10. Micic D, Stankovic S, Lalic N, Dukic V, Polovina S. Prognostic Value of Preoperative Neutrophil-to-Lymphocyte Ratio for Prediction of Severe Cholecystitis. Journal of Medical Biochemistry. 2017;37:121-7.
11. Naess A, Mo R, Nilssen SS, Eide GE, Sjursen H. Infections in patients hospitalized for fever as related to duration and other predictors at admittance. Infection. 2013;42:485-92.
12. Varkila MRJ, Marrec L, Daix T, Hoefer IE, Haitjema S, Bonten MJM, et al. Persistent lymphocytopenia does not increase nosocomial infection risk in the icu. American Journal of Respiratory and Critical Care Medicine. 2021;203:913-6.

**Not in English (n = 4)**

1. Che-Morales JL, Cortes-Telles A. Neutrophil-to-lymphocyte ratio as a serum biomarker associated with community acquired pneumonia. Revista Medica del Instituto Mexicano del Seguro Social. 2019;56(6):537-43.
2. Conlledo R, Rodriguez A, Godoy J, Merino C, Martinez F. Total globulins and lymphocyte count as markers of mortality in sepsis and septic shock. Revista Chilena de Infectologia. 2012;29:192-9.
3. Gonzalez Mendez A, Maevskaya M, Zharkova M, Lunkov V, Tsvetaeva E, Ivashkin V. Neutrophil to lymphocyte ratio as predictor of systemic inflammatory response syndrome and mortality in patients with decompensated liver cirrhosis. United European Gastroenterology Journal. 2019;6:A348.
4. Lorente L, Martin MM, Ortiz-Lopez R, Alvarez-Castillo A, Ruiz C, Uribe L, et al. Association between neutrophil-to-lymphocyte ratio in the first seven days of sepsis and mortality. Enfermedades Infecciosas y Microbiologia Clinica. 2015;40:235-40.
